# Injectable sustained‐release poly(lactic‐co‐glycolic acid) (PLGA) microspheres of exenatide prepared by supercritical fluid extraction of emulsion process based on a design of experiment approach

**DOI:** 10.1002/btm2.10485

**Published:** 2023-01-02

**Authors:** Heejun Park, Eun‐Sol Ha, Jeong‐Soo Kim, Min‐Soo Kim

**Affiliations:** ^1^ College of Pharmacy Duksung Women's University Seoul South Korea; ^2^ College of Pharmacy Pusan National University Busan South Korea; ^3^ Dong‐A ST Research Institute Dong‐A ST Co. Ltd. Giheung‐gu Yongin‐si Gyeonggi South Korea

**Keywords:** Box–Behnken design, exenatide, pharmacodynamic, pharmacokinetic, PLGA microspheres, supercritical fluid extraction of emulsions, sustained‐release

## Abstract

This study aimed to develop an improved sustained‐release (SR) PLGA microsphere of exenatide using supercritical fluid extraction of emulsions (SFEE). As a translational research, we investigated the effect of various process parameters on the fabrication of exenatide‐loaded PLGA microspheres by SFEE (ELPM_SFEE) using the Box–Behnken design (BBD), a design of experiment approach. Further, ELPM obtained under optimized conditions and satisfying all the response criteria were compared with PLGA microspheres prepared using the conventional solvent evaporation (ELPM_SE) method through various solid‐state characterizations and in vitro and in vivo evaluations. The four process parameters selected as independent variables were pressure (*X*_1_), temperature (*X*_2_), stirring rate (*X*_3_), and flow ratio (*X*_4_). The effects of these independent variables on five responses, namely the particle size, its distribution (SPAN value), encapsulation efficiency (EE), initial drug burst release (IBR), and residual organic solvent, were evaluated using BBD. Based on the experimental results, a desirable range of combinations of various variables in the SFEE process was determined by graphical optimization. Solid‐state characterization and in vitro evaluation revealed that ELPM_SFEE improved properties, including a smaller particle size and SPAN value, higher EE, lower IBR, and lower residual solvent. Furthermore, the pharmacokinetic and pharmacodynamic study results indicated better in vivo efficacy with desirable SR properties, including a reduction in blood glucose levels, weight gain, and food intake, for ELPM_SFEE than those generated using SE. Therefore, the potential drawback of conventional technologies such as the SE for the preparation of injectable SR PLGA microspheres could be improved by optimizing the SFEE process.

## 1 INTRODUCTION

Polymer‐drug microspheres are the most promising drug delivery systems for controlled release device development, especially if produced with a narrow particle size distribution (PSD).^1^ The release kinetics of sustained‐release (SR) microspheres are mainly governed by particle size, surface morphology, and inner structure, as well as drug and polymer physicochemical properties.^2^, ^3^, ^4^ In particular, microsphere particle size and its distribution determine the surface area/volume ratio and, thereby, the surface available for drug release. Monodispersed microspheres may exhibit the most uniform drug release, avoiding peak‐related side effects.^5^, ^6^, ^7^

Among the various microsphere production technologies, emulsion‐based particle formation is the most widely used because of its unique advantages, including the uniform generation of small spherical particles and higher encapsulation efficiency (EE). This emulsion‐based micro/nanoparticle manufacturing process can be applied for mass production, but products manufactured using this process need to meet the quality criteria for medicinal products. Maintenance of such critical quality parameters necessitates suitable residual organic solvents, along with a uniform size distribution in the desired particle size range, pore size, and surface area. One of the most important factors for this emulsion‐based micro/nanoparticle technology is the extraction efficiency associated with rapid removal of the organic solvent. Microsphere fabrication through solvent removal from emulsion is conventionally performed using three basic techniques: solvent evaporation (SE) or extraction, phase separation (coacervation), and spray drying.^3^, ^8^, ^9^, ^10^, ^11^ Most of the recently suggested solvent removal processes for emulsions have been developed by modifying these three basic processes. Although these technologies are being studied over 50 years, large batch variations, low EE, and increased risk of side effects owing to excessive initial drug burst release (IBR) are commonly observed. Hence, development of improved solvent removal methods to produce more uniform and reproducible particles is still needed.^12^, ^13^, ^14^

Considering this, a technology called supercritical fluid extraction of emulsions (SFEE), which uses the unique mass transfer mechanism and solvent power of a supercritical fluid (SCF), has been proposed to overcome the shortcomings of conventional technologies including SE, extraction, and spray drying.^15^, ^16^, ^17^, ^18^, ^19^, ^20^

Supercritical fluid solvents are an attractive alternative to incompressible organic liquid solvents because they possess a liquid‐like dissolving power while exhibiting the transport properties of a gas. Near the critical point, small changes in temperature or pressure can produce large changes in the density/solvation ability of fluids; the lower viscosity and higher diffusivity of SCFs with respect to liquid solvents improve mass transfer, which is often a limiting factor for extraction processes.^21^, ^22^, ^23^, ^24^ Owing to their favorable properties, SCFs (mainly supercritical carbon dioxide, SC‐CO_2_) have been proposed for a wide range of extraction applications.^25^, ^26^, ^27^, ^28^, ^29^

Recently, supercritical fluid extraction (SFE) has been proposed for use in manufacturing PLGA microspheres, starting from emulsions. In particular, SC‐CO_2_ has been proposed as an extraction agent for the “oily” phase (organic solvent phase) of emulsions to produce solvent‐free microspheres.^30^, ^31^, ^32^ This process, called SFEE produces an aqueous suspension of PLGA microspheres after supercritical extraction of the organic solvent in the emulsion. PLGA microsphere aggregations are not observed in the SFEE process, because of the presence of the external water phase, which is immiscible with SC‐CO_2_ and prevents their aggregation.^33^ The SFEE process is also credited to be rapid, owing to the enhanced mass transfer of SC‐CO_2_, and is capable of affecting the size distribution of the prepared microspheres; overall, the fast extraction rate results in a narrow PSD because droplet aggregation is minimized.^34^

The SFEE process proposed to solve several problems of conventional extraction process can improve the pharmaceutical properties of medicinal microspheres by exploiting the inherent advantages of SCF to achieve superior solvent removal efficiency and rapid solidification. Therefore, SFEE is expected to be an excellent technology for manufacturing superior‐quality solid microspheres compared to those obtained using the existing particle manufacturing technologies. Owing to the mild process temperature, SFEE can be the first choice for biopharmaceutical particle manufacturing, on considering stability.^35^, ^36^, ^37^

Currently, extraction technologies using SCF are being applied commercially in the food industry to extract effective active ingredients from natural raw materials or to selectively remove unwanted substances.^38^, ^39^, ^40^, ^41^ In the food industry, many experimental design‐based studies have been undertaken for a fundamental understanding of the correlation between process parameters and output, thereby completing the optimization, scale‐up, and validation of the SFE process, and successful commercialization has been achieved based on this fundamental understanding.^42^, ^43^, ^44^ In contrast to the food science field, control of particle size, distribution, and drug loading by varying the SC‐CO_2_ process conditions has been explored in the pharmaceutical field,^45^, ^46^, ^47^, ^48^, ^49^, ^50^ but only a few studies was scarcely reported for the effects of various process variables of SFEE on the output. Further, its application range and cases are very limited despite reports that the SFEE process has many advantages for the preparation of microspheres containing bioactive materials.

Thus, the purpose of this study was to develop an improved SR PLGA microsphere of exenatide using SFEE. Exenatide, the first incretin‐mimetic glucagon‐like peptide‐1 (GLP‐1) receptor agonist that has been approved as an adjunct therapy for type‐2 diabetes mellitus (T2DM) by subcutaneous (SC) injection, was used as a model therapeutic biological material (with 39‐amino acids, MW 4186.6 g/mol).^51^, ^52^, ^53^, ^54^ The SR exenatide delivery system including Bydureon™ or Byetta® Long‐Acting Release (LAR) was developed by Amylin Pharmaceuticals/Eli Lilly using Alkermes's water‐in‐oil‐in‐oil (W/O/O) coacervation technology.^55^ Although these SR injections have been successfully developed commercially, they have been reported to have several limitations. It was reported that the mean particle size of Bydureon™ microspheres is approximately 50 μm, which requires the use of painfully large, 23‐gauge needles for SC injection, thus decreasing patient compliance.^56^, ^57^, ^58^, ^59^ In particular, the weekly dose of exenatide in Byetta® LAR microspheres (i.e., single injection of 2 mg/human) is much higher (14–28 fold) than that of Byetta® (i.e., twice‐daily injection of 70–140 ug/human). From this fact, it was suggested that the improper release of exenatide from Byetta® LAR in vivo may explain the necessity for a much higher dose.^60^ Another study reported that the drug burst release was high (approximately 45%) when they prepared microspheres following the commercialized ELPM formulation.^59^ These problems can increase the risk of adverse effects due to overdose of exenatide.^54^ It was also reported that in vivo sustained PK profile of Byetta® LAR showed poor SR characteristics with low release between days 4 and 17 in animal PK study.^55^ In addition, the commercial coavervation method for the fabrication of PLGA microsphere has several problems such as rather complicated process and long manufacturing time with multiple process steps, the use of additional oil‐based dispersion solvents and toxic solvents such as n‐hexane to remove other solvent. Various pharmaceutical approaches have been tried to overcome these problems. In particular, several research groups have been attempted to reduce burst release and the incomplete release of exenatide from commercial SR microsphere products.^54^, ^55^, ^57^, ^59^, ^61^, ^62^, ^63^, ^64^, ^65^, ^66^, ^67^, ^68^, ^69^, ^70^, ^71^, ^72^, ^73^, ^74^, ^75^, ^76^ Nevertheless, limitations in the microsphere manufacturing process, physical quality characteristics and drug release property have not been fully overcome.

Fundamental ways to overcome this limitation are to simultaneously try approaches to improve the pharmaceutical properties of SR injection formulations as well as the improvement or development of manufacturing processes for its commercialization. These attempts are not only limited to basic research on a laboratory scale but can drive and provide improved innovative pharmaceutical technology that can impact clinical practice and/or commercial SR injection formulations. Thus, we also investigated the effect of various process parameters on the fabrication of exenatide‐loaded PLGA microspheres by SFEE (ELPM_SFEE) using the Box–Behnken design (BBD), a design of experiment (DOE), as a translational research approach to reduce the gap between fundamental understanding of the SFEE process and its application to scale‐up manufacturing of PLGA microsphere for commercialization. To the best of our knowledge, there have been no attempts to investigate the SFEE process for the production of SR microsphere containing exenatide based on the DOE approach. BBD was used as a response surface methodology (RSM) to mathematically and statistically determine the patterns and relationships between independent variables, including the SFEE process parameters (pressure, temperature, and stirring rate) and important responses related to the fabrication of PLGA microspheres, such as EE, PSD, IBR, and residual organic solvent. In addition, to evaluate critical pharmaceutical quality properties and clinical relevance as a final drug product, various in vitro characterizations, such as scanning electron microscopy (SEM), particle size analysis, drug release, Brunauer–Emmett–Teller (BET) specific surface area measurement, water vapor sorption analysis, Fourier‐transform infrared (FT‐IR) spectroscopy, circular dichroism (CD) spectroscopy, and evaluation of EE, were also performed in comparison with ELPM prepared by the SE process, which is a common conventional commercial technique for preparing SR microsphere formulations. Further, the feasibility of clinical use for the PLGA microsphere formulation prepared using the optimized SFEE process was evaluated. Plasma exenatide concentrations as a pharmacokinetic (PK) property, as well as nonfasting blood glucose, and changes in food intake and body weight as pharmacodynamic (PD) and therapeutical efficacy properties were evaluated in streptozotocin (STZ)‐induced diabetic mice following treatment with this novel formulation. The results of these evaluations were compared with those of PLGA microspheres prepared using the conventional SE method.

## 2 MATERIALS AND METHODS

### 2.1 Materials

Exenatide was used as a model therapeutic biological material (American Peptide Company Inc., USA). PLGA 50/50 was gifted by Boehringer Ingelheim (Resomer 503H, MW 24,000–38,000, Germany). Sucrose, lysine, proline, and polyvinyl alcohol (PVA, MW 13,000–23,000) were obtained from Sigma Aldrich (USA). β‐Cyclodextrin was supplied by ISP Technologies, Inc. (Wayne, NJ, USA). Dichloromethane (DCM), dimethylformamide (DMF), acetone, acetonitrile (ACN), tetrahydrofuran (THF), and trifluoroacetic acid (TFA) were obtained from Merck (Fair Lawn, New Jersey, USA). All other chemicals were of reagent grade and were used without further purification.

### 2.2 Reverse phase high performance liquid chromatography for exenatide quantification

Reverse phase high performance liquid chromatography (RP–HPLC) was performed using an Agilent 1290 Infinity HPLC system (Germany) to quantitatively analyze the exenatide content, as previously described, with slight modifications.^55^, ^77^, ^78^ The HPLC system comprised a high‐pressure pump (Model 1260 Quat Pump VL) and an auto sampler (Model 1260 ALS) that was maintained at a constant temperature of 5°C. Exenatide was separated using an octadecylsilane column (Kintex C18, 2.6 μm, 100 Å, 4.6 × 100 mm^2^, Phenomenex Inc., USA) at a temperature of 30°C. Exenatide was detected at 210 nm using a UV detector (Model 1260 VWD DL). The mobile phase comprised 40% ACN with 0.1% TFA/60% water with 0.1% TFA, eluted at a flow rate of 1.5 ml/min, and the injection volume was set as 10 μl. The calibrated linear range of exenatide quantification method using HPLC was 2.5 ~ 200 ug/ml (R^2^ > 0.999).

### 2.3 Preparation of exenatide loaded PLGA microspheres

#### 2.3.1 Preparation of W/O/W double emulsion

The formulation used in this study was determined based on the results of our previously reported studies,^54^, ^74^, ^77^, ^78^ with minor modifications. First, 30 mM acetate buffer (pH 4.5) was prepared and exenatide (10 mg), sucrose (40 mg), β‐cyclodextrin (5 mg), and proline (0.05 M) were dissolved in 0.1 ml of the buffer. An aqueous solution was used as the inner water phase. The oil phase was prepared by dissolving 185 mg of PLGA in 2.5 ml DCM. To prepare a W/O primary emulsion, the inner water phase was carefully poured into the oil phase, stored in an ice bath, and immediately emulsified using a homogenizer (T‐18 Basic ULTRA‐TURRAX®, IKA®‐WERKE GMBH & Co. KG, Germany) for 5 min at 21,500 rpm. Finally, to complete the preparation with a W/O/W double emulsion, the W/O primary emulsion was added to 1% PVA with 0.1 M lysine (12 ml) as the outer water phase, and then emulsified using a homogenizer (T‐18 Basic ULTRA‐TURRAX®, IKA®‐WERKE GMBH & Co. KG, Germany) for 1 min at 6500 rpm. After homogenization, an additional 12.5 ml of 1% PVA with 0.1 M lysine was slowly poured into the double emulsion and gently mixed to obtain system equilibrium.

#### 2.3.2 SFEE process

**FIGURE 1:**
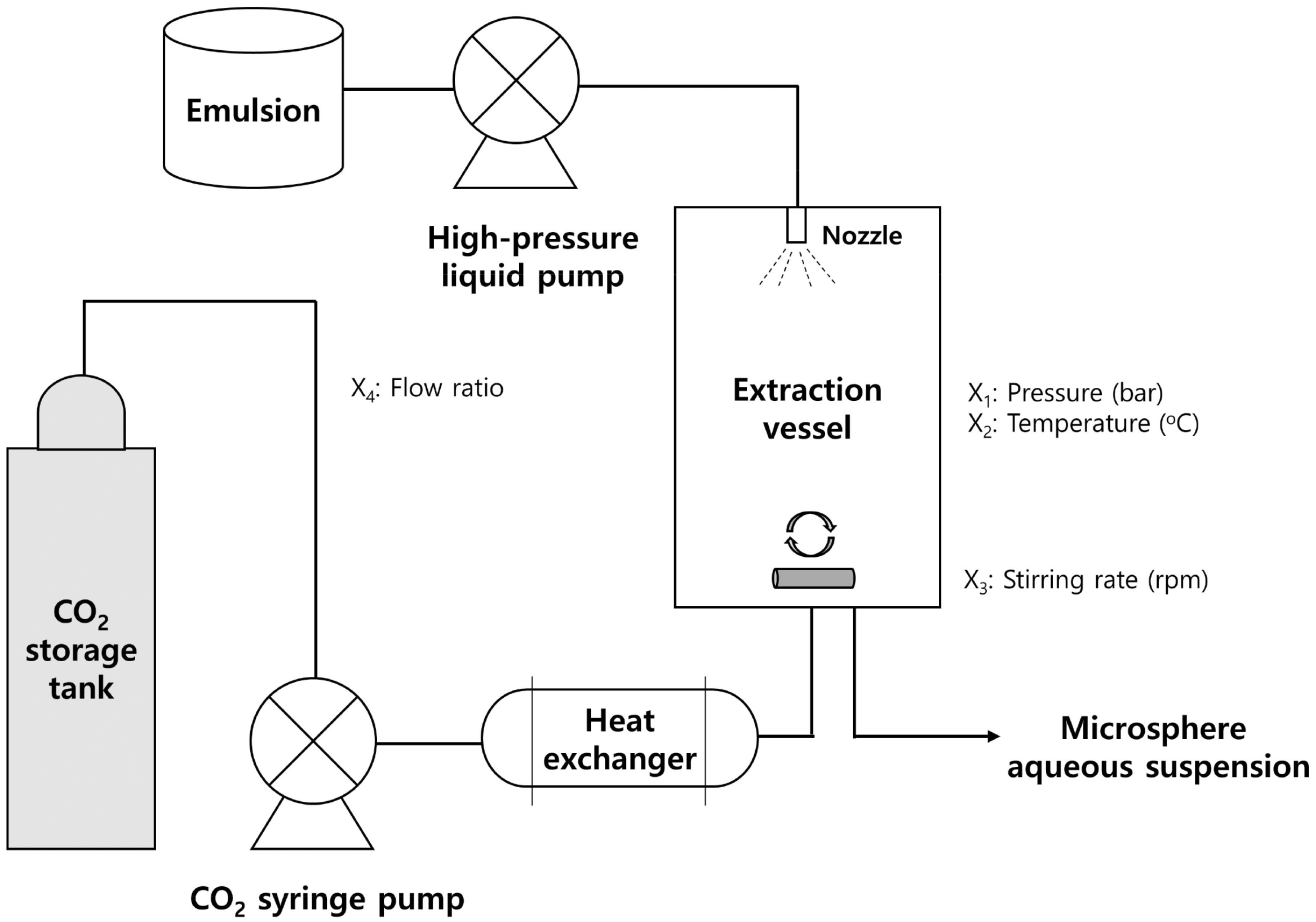
Schematic representation of the supercritical fluid extraction of emulsions (SFEE) apparatus. (Adopted with permission by Creative Common CC BY license).^64^

PLGA microspheres were prepared using the SFEE process under various process conditions to assess the effect of process variables on the properties of PLGA microspheres. A schematic representation of the SFEE apparatus used for microsphere preparation is shown in Figure 1.^79^

First, liquid CO_2_ from the storage tank was delivered into the preheater tank; heated and compressed CO_2_ was then delivered to the cylindrical stainless‐steel extraction vessel (approximately 70 ml) through the spray nozzle using an ISCO syringe pump (Model 260D, USA) until the desired pressure was reached. Once the pressure and temperature were equilibrated under the desired conditions, 15 ml of the W/O/W emulsion was injected into the high‐pressure extraction vessel using an HPLC liquid pump (Model 307; Gilson Inc., USA). Thereafter, SC‐CO_2_ continued to flow into the vessel at a constant flow rate to extract DCM from the double emulsion using a backpressure regulator (Tescom, model 26‐1723‐24‐194, USA) at a constant pressure and temperature. The emulsion was stirred using a magnetic stirrer during extraction for effective diffusion and to prevent coalescence in the emulsion. Each SFEE process was performed for 5 h. After complete the extraction, the vessel was slowly depressurized to atmospheric pressure. The collected PLGA microsphere suspensions were then washed using a sterilized injection solution (0.9% NaCl and 5% dextrose) by centrifugation at 600× g (Eppendorf centrifuge 5019R, Germany) followed by supernatant removal. PLGA microsphere suspension was washed thrice using sterile deionized water for performing solid‐state characterization which will be described in Section 2.5. Finally, the washed wet PLGA microspheres were frozen in a deep freezer and then freeze‐dried (FD8508, Ilshin BioBase Co. Ltd., Gyeonggi‐do, Korea) at a vacuum of 50 mTorr.

#### 2.3.3 Experimental design and optimization of the SFEE process for manufacturing microspheres using BBD

The effect of critical process parameters in SFEE, alone or in combination, as independent variables on five responses, including the particle size and its distribution (in terms of SPAN value), EE, IBR, and residual organic solvent, were elucidated using BBD using RSM. These responses were selected as critical quality attributes for SR PLGA microspheres based on previously reported literature.^1^, ^2^, ^3^, ^4^, ^5^, ^6^, ^7^ Further, based on the results from our preliminary study, the CQA, which can be affected by the SFEE process variables, was also considered for the proper response selection.

For BBD, the experimental methodology and generation of mathematical models were performed using Design Expert software (version 7.0, Stat‐Ease Inc., Minneapolis, MN, USA). A BBD with four factors, three levels, and a total of 27 experimental runs, including three center points per block, was selected for RSM. The four process parameters used as independent variables were pressure (*X*_1_), temperature (*X*_2_), stirring rate (*X*_3_), and flow ratio (*X*_4_). These chosen factors are well known as critical process parameters for SFE processes in various fields.^17^, ^80^, ^81^, ^82^, ^83^, ^84^, ^85^, ^86^, ^87^ The levels of these four process parameters were determined based on a preliminary study conducted with a wider range of the four independent variables (*X*_1_: 30–65 °C, *X*_2_: 70–160 bar, *X*_3_: 50–1000 rpm, *X*_4_: 1–40). Here, the flow ratio indicates the ratio of the CO_2_ flow rate to the emulsion injection rate (1 ml/min). The process conditions of 35°C, 85 bar, 500 rpm with a flow ratio of 5 were estimated as suitable from the preliminary study, and were set as the center points for the current study. To examine their effect on the response in detail by fine control of process parameters as independent variables within a narrow condition range and to optimize the process based on these conditions, the levels of three process parameters for BBD in this study were set up as presented in Table 1. The experimental design comprised a set of points lying at the midpoint of each edge, with three replicates of the center point from the multidimensional cube. Except for the center point, all experimental runs were conducted in triplicate, and the average values were used for the BBD.

**TABLE 1:** Independent variables and responses selected for the Box–Behnken design (BBD) and optimization study

Factors	Levels		
	Low (−1)	0	High (+1)
*X*_1_: Pressure (bar)	75	85	95
*X*_2_: Temperature (°C)	30	35	40
*X*_3_: Stirring rate (rpm)	300	500	700
*X*_4_: Flow ratio	1	5	9

Responses	Acceptable range		Target
	Low limit	High limit	
*Y*_1_: Particle size (μm)	20	30	In range
*Y*_2_: SPAN	0	1.3	In range
*Y*_3_: Encapsulation efficiency (EE, %)	85	100	In range
*Y*_4_: Initial burst release (IBR, %)	3.3	10	In range
*Y*_5_: Residual solvent (ppm)	0	150	In range

The values of independent variables and their responses are given in Table 2. A randomized sequence of actual experiments was used to minimize the unpredicted variability caused by irrelevant variables in responses. The experimentally obtained data were analyzed by multiple regression analysis, and four mathematical models (linear, 2FI, quadratic, and cubic) were used for simultaneous fitting to various responses. Analysis of variance (ANOVA) was used for statistical justification by evaluating the significance of SFEE process factors as independent variables on the responses and checking the quality of the fitted polynomial model (Table 2). Based on the ANOVA results, the most adequate model was selected, and the fit statistics for the model were obtained. Larger *F*‐values and smaller *p*‐values indicated higher significance of the model.^87^, ^88^, ^89^ A *p*‐value less than 0.05 and 0.01 indicates that the model terms are statistically significant at the 95% confidence level and are highly significant at the 99% confidence level.^90^, ^91^, ^92^ Thus, *p* values <0.05 obtained from ANOVA were considered statistically significant. In contrast, a *p*‐value greater than 0.05 indicated that the model terms were not significant.^93^

**TABLE 2:** Box–Behnken experimental design: process parameters as independent variables, measured responses and summary of model fitting and statistical analysis.

Run	Factors				Responses				
	*X*_1_	*X*_2_	*X*_3_	*X*_4_	*Y*_1_	*Y*_2_	*Y*_3_	*Y*_4_	*Y*_5_
	Pressure (bar)	Temperature (°C)	Stirring rate (rpm)	Flow ratio	Particle size (μm)	SPAN	Encapsulation efficiency (EE, %)	Initial burst release (IBR, %)	Residual solvent (ppm)
1	75	30	500	5	21.7	1.16	60.36	17.91	217.2
2	85	30	500	1	26.6	1.32	70.30	12.42	176.8
3	85	40	300	5	31.5	1.73	61.88	11.44	55.4
4	85	30	300	5	28.6	1.47	68.00	11.18	176.9
5	85	35	500	5	21.3	1.18	83.88	9.31	79.7
6	75	35	700	5	21.5	1.18	60.86	17.60	129.1
7	85	35	700	9	23.3	1.25	78.50	12.50	60.1
8	95	30	500	5	23.4	1.32	71.72	16.39	126.5
9	95	35	500	9	24.2	1.31	67.50	15.10	45.3
10	85	35	300	9	25.6	1.36	72.90	9.10	69.8
11	95	35	300	5	29.7	1.62	56.12	14.32	74.2
12	75	35	500	9	23.0	1.21	58.50	16.10	132.5
13	85	30	500	9	23.9	1.27	75.50	11.50	112.7
14	85	30	700	5	23.3	1.23	76.58	14.93	144.8
15	85	35	700	1	25.1	1.3	76.60	13.50	202.7
16	85	40	700	5	25.9	1.51	63.26	16.48	46.5
17	85	35	500	5	23.5	1.23	85.56	8.24	84.7
18	95	35	700	5	25.7	1.32	53.90	19.04	43.7
19	95	40	500	5	27.3	1.65	50.44	19.70	45.9
20	75	35	300	5	29.9	1.58	48.44	15.90	164.8
21	85	40	500	9	27.2	1.41	72.50	11.10	47.8
22	85	35	300	1	28.8	1.49	65.00	10.50	208.4
23	75	35	500	1	28.1	1.42	50.40	16.37	243.8
24	85	40	500	1	29.4	1.59	60.20	14.26	137.5
25	85	35	500	5	24.8	1.21	88.12	7.90	91.8
26	75	40	500	5	24.5	1.33	73.44	12.66	157.3
27	95	35	500	1	28.3	1.48	60.10	17.80	122.1
28	75	30	500	5	21.7	1.16	60.36	17.91	217.2
29	85	30	500	1	26.6	1.32	70.30	12.42	176.8

Response	Model (*p*‐value)	Lack of fit	R^2^	R^2^ pred	R^2^ adj	Adequate Precision
*Y*_1_: Particle size (μm)	<0.0001	0.5885	0.6272	0.5785	0.5191	11.4400
*Y*_2_: SPAN	<0.0001	0.1216	0.8826	0.8305	0.7359	13.2976
*Y*_3_: Encapsulation efficiency (EE, %)	<0.0001	0.1472	0.8591	0.7845	0.5473	12.0675
*Y*_4_: Initial burst release (IBR, %)	<0.0001	0.3713	0.9382	0.9055	0.7953	18.1410
*Y*_5_: Residual solvent (ppm)	<0.0001	0.0663	0.8907	0.8647	0.8202	20.3836
*Y*_1_ = 23.2002 + 0.3050 *X*_2_–0.0122 *X*_3_–0.3979 *X*_4_						
*Y*_2_ = 12.4147–0.1171 *X*_1_–0.3235 *X*_2_–0.0039 *X*_3_–0.0488 *X*_4_ + 0.0007 *X*_1_^2^ + 0.0050 *X*_2_^2^ + 0.0000 *X*_3_^2^ + 0.032 *X*_4_^2^						
*Y*_3_ = −2188.2344 + 37.8041 *X*_1_ + 33.8390 *X*_2_ + 0.2626 *X*_3_ + 5.0385 *X*_4_–0.1718 *X*_1_ *X*_2_–0.1866 *X*_1_^2^–0.2845 *X*_2_^2^–0.0002 *X*_3_^2^–0.4147 *X*_4_^2^						
*Y*_4_ = 708.3059–11.8670 *X*_1_–10.6523 *X*_2_–0.0429 *X*_3_–1.0081 *X*_4_–0.0428 *X*_1_ *X*_2_ + 0.0613 *X*_1_^2^ + 0.1005 *X*_2_^2^ + 0.0001 *X*_3_^2^ + 0.0811 *X*_4_^2^						
*Y*_5_ = 2166.6428–33.7651 *X*_1_–7.7417 *X*_2_–28.0574 *X*_4_ + 0.1698 *X*_1_^2^ + 1.5076 *X*_4_^2^						

Further, lack of fit, coefficient of variance (CV, %), coefficient of determination (R^2^), adjusted coefficient of determination (R^2^ adj), predicted coefficient of determination (R^2^ pred), adequate precision, and prediction sum of square (PRESS) of the models were also used to determine the adequacy and goodness of fit for each polynomial equation model.^47^, ^94^, ^95^, ^96^ Among the different polynomial models, the best‐fit mathematical model was selected based on statistical parameters. The polynomial quadratic equation for the model is given as:

(1) $$Y=A_{0}+\sum A_{i}X_{i}+\sum A_{\mathrm{ij}}X_{i}X_{j}+\sum A_{\mathrm{ii}}X_{i}^{2}$$

where *Y* is the response; *X*_*i*_ *–X*_*j*_ are independent variables corresponding to pressure, temperature, stirring rate, and flow ratio, respectively; *X*_*i*_ *X*_*j*_ and *X*_*i*_^2^ are the interaction and quadratic terms, respectively; *A*_0_ is the constant term of the model intercept coefficient (which indicates the overall average response of all runs); and *A*_*i*_, *A*_*ii*_, and *A*_*ij*_ is the regression coefficient of the linear, quadratic and interaction terms, respectively. Significant model terms are included in the equation.

To visualize the relationships between responses and independent variables and to deduce the optimal conditions, the fitted quadratic polynomial equation was expressed as a contour plot. The selected best‐fitting model was used to optimize the SFEE process for preparing PLGA microspheres with the most desirable target response value. PLGA microspheres were prepared under the predicted optimized SFEE process conditions, and all dependent variables were experimentally obtained.

#### 2.3.4 Solvent evaporation process

PLGA microspheres were also prepared using the SE process at a condition reported previously for comparison with the SFEE process.^77^, ^78^ The emulsion preparation process was identical to that for SFEE. The DCM in the double emulsion was removed using a rotary vacuum evaporator (N‐1110, EYELA, USA) at 35°C and 150 rpm for 7 h. The resulting microspheres were prepared as dried formulations using the washing and freeze‐drying processes described for SFEE.

### 2.4 Solid‐state characterization of PLGA microspheres

#### 2.4.1 Scanning electron microscopy

The morphology of the prepared PLGA microspheres was examined by SEM (JSM‐6700F, JEOL, Japan). The dried microsphere powder samples were spread on a metal support stub to which double‐sided conductive carbon adhesive tape was attached; the excess powder was removed using a nitrogen gas spray gun. The microspheres were then coated with gold–palladium using an automated sputter coater. SEM analysis was performed at an accelerating voltage of 5 kV, and several images were collected at appropriate magnification.

#### 2.4.2 Particle size analysis

The particle size of the prepared PLGA microspheres was measured using a Mastersizer 2000 (Malvern Instruments, Malvern, UK), based on a laser diffraction method. The volume mean diameter (VMD, μm) was obtained as the representative value of particle size. Further, the PSD was estimated in terms of the SPAN value. The SPAN value was calculated by dividing *D*_90%_ – *D*_10%_ by *D*_50%_; generally, a smaller SPAN value means a narrower PSD.

#### 2.4.3 Brunauer–Emmett–Teller specific surface area

The specific surface area of the prepared blank PLGA microspheres without exenatide was analyzed using TriStar II 3020 (Micromeritics, GA, USA). The sample powders were loaded into standardized sample tubes, and precisely weighed. After completion of degassing procedure using a FlowPrep 060 degasser (Micromeritics, GA, USA), the nitrogen adsorption–desorption isotherm was determined at −196°C. The specific surface area was determined by applying the adsorption–desorption isotherm results to BET theory.

#### 2.4.4 Water vapor sorption analysis

The moisture sorption/desorption behavior was evaluated using a dynamic vapor sorption (DVS, Surface Measurement Systems, UK) apparatus equipped with a microbalance to measure mass changes (±0.1 μg) under controlled temperature and humidity, maintained by nitrogen gas flow through a humidification stage. Powder samples of PLGA microspheres were loaded onto an aluminum pan and inserted into a humidity/temperature control chamber under atmospheric conditions. An empty pan was used as the reference. Then, prior to actual measurements, it was equilibrated to 0% relative humidity (RH) at 25°C. The experimental cycle comprised a sorption cycle, followed by a desorption cycle. The instrument was programmed for moisture sorption from 0% to 95% RH in 5% RH steps at 25°C. For the moisture desorption isotherm, the RH was programmed from 95% to 0% RH in 5% RH steps at 25°C. The weight change was measured at each programmed RH step when it reached equilibrium conditions set to <0.01% of total mass change within 15 min.

### 2.5 Secondary structure and conformational stability of exenatide in PLGA microspheres

#### 2.5.1 Circular dichroism

Circular dichroism spectroscopy was conducted on a Jasco J‐810 spectrometer (Jasco, Japan) using a 0.1 cm path cuvette with a scan rate of 50 nm/min at 30°C. For extracting exenatide from solid PLGA microspheres, microspheres equivalent to 10 mg exenatide were dissolved in 3 ml acetonitrile using a vortex mixer; then, 3 ml of acetate buffer solution (pH 4.5, 30 mM) was added and immediately vortexed to precipitate the PLGA polymer. The suspension obtained after acetonitrile removal by rotary evaporation under vacuum was centrifuged (1730 MR, GYROZEN Co. Ltd., Korea) at 12,000 rpm for 5 min to separate the precipitated PLGA polymer. The collected supernatant was used as the sample solution for CD spectroscopy. For comparing secondary structural integrity, raw exenatide solution (10 mg/3 ml) in acetate buffer (pH 4.5, 30 mM) was used as a control. A total of 24 scans were performed, and the averaged spectrum was used for secondary structural analysis.

#### 2.5.2 Fourier‐transform infrared spectroscopy

Fourier‐transform infrared spectroscopy was performed on a Nicolet 380 FT‐IR spectrometer (Thermo Fisher, USA) equipped with an attenuated total reflectance (ATR) accessory. The sample solution used for CD spectroscopy was also used for FT‐IR analysis. Prior to actual measurement, background spectra were obtained under nitrogen gas purging of the detector to minimize interference from other gases such as water vapor and/or CO_2_. The spectral range collected was 400–4000 cm^−1^. The resolution and scan number were 4 cm^−1^ and 256, respectively. For analyzing peptide secondary structure, FT‐IR spectra were processed to the second derivative using OriginPro 8 software with 7‐point smoothing (OriginLab Corp., Northampton, MA, USA) as reported earlier.^77^, ^97^

### 2.6 Residual solvent on PLGA microspheres

The residual solvent concentrations (in ppm) on PLGA microspheres prepared using the SFEE and SE methods were measured with the GC method. Accurately weighed lyophilized PLGA microspheres were dissolved entirely in THF (1 ml). Subsequently, 4 ml of methanol was added and the mixture was vortexed for 1 min to precipitate the PLGA polymer. To remove the precipitated PLGA, samples were centrifuged (1730 MR, GYROZEN Co. Ltd., Daejeon, Korea) at 12,000 rpm for 10 min. For analyzing the residual solvent, the collected supernatant was injected into the GC system under the same analysis conditions as described above. The residual solvent amount obtained by GC analysis was similar to that obtained by analyzing the amount of residual solvent in the suspension before freeze‐drying. Therefore, the amount of residual organic solvent directly analyzed from lyophilized microspheres was used in this study.

### 2.7 Drug encapsulation efficiency

The sample solution used in CD spectroscopy was also used to determine EE%. The sample solutions were injected into the RP‐HPLC system for exenatide quantification, as described above. The EE values were obtained using Equation (2):

(2) $$\mathrm{EC}\;\left(\%\right)=\left(\text{Measured}\;\mathrm{LC}/\text{Theoretical}\;\mathrm{LC}\right)\times 100$$

where LC is the loading capacity % obtained by multiplying the mass of exenatide in the microspheres divided by the mass of the microspheres by 100. A linearly proportional correlation between EE obtained using an indirect method, by determining the amount of drug present in the aqueous phase before freeze‐drying, and EE obtained by direct extraction from microspheres was confirmed. Accordingly, the EE value directly analyzed from the microspheres after freeze‐drying was used in this study.

### 2.8 In vitro drug release test

The drug release medium was prepared by dissolving tween 20 (0.02% w/v) and sodium azide (0.01% w/v) in pH 7.4 phosphate‐buffered saline (PBS), as a dispersing and a preserving agent, respectively. Briefly, 20 mg of PLGA microspheres were accurately weighed and introduced into 2 ml of drug release medium in a glass tube. After starting incubation in a shaking water bath at 37°C and 100 rpm, the medium was collected at predetermined time points and a corresponding volume of medium was newly replenished. The withdrawn medium was collected in microcentrifuge tubes (Eppendorf) and centrifuged (12,000 rpm, 5°C, 10 min) to separate undissolved material; the clear supernatant was the used for exenatide quantification using the RP‐HPLC method. The IBR indicates the percentage of exenatide released on the first day.

### 2.9 In vivo study in STZ‐induced diabetic mice

#### 2.9.1 In vivo pharmacokinetic study

The in vivo PK study in STZ‐induced diabetic mice was performed using a previously reported experimental method. Male ICR mice (Samtaco Biokorea, Inc., Seoul, Korea) aged 8 weeks and weighing 30–31 g were housed in stainless steel cages with food and water ad libitum in a clean room under a 12 h/12 h light/dark cycle at a controlled temperature and RH of 21.5 ± 1.5°C and 50%, respectively. All animal experiments were approved by the Ethics Committee of KPC Laboratory (No. I‐1703060, Gwangju, Korea) and implemented in compliance with the institutional guidelines for the care and use of laboratory animals.

Streptozotocin was used to induce diabetes, with slight modifications to a previously reported method.^78^, ^98^, ^99^, ^100^ After acclimation for a week, mice were intraperitoneally injected with a solution of STZ dissolved in 0.05 M citrate buffer (pH 4.5) at a dose of 120 mg/kg. Blood glucose levels were measured (AccuCheck Active, Roche Diagnostics, Germany) on the third day after STZ intraperitoneal injection, and mice with levels higher than 300 mg/dl were chosen as diabetes mellitus (DM) mouse models for the PK and PD studies.

Animals were divided into two groups, which were administered exenatide‐loaded PLGA microsphere formulations prepared using SE and the optimized SFEE process, respectively. Each group was housed in cage including six STZ‐induced DM mice. All animals were given free access to tap water and a normal diet.

Prior to administering the formulation, an aqueous suspension containing exenatide‐loaded PLGA microspheres (ELPMs) was prepared by uniform dispersion of dried PLGA microspheres in an aqueous dispersion medium of 0.87% NaCl, 1.5% Na CMC, and 0.1% Tween 20. The amount of microspheres used to prepare the suspension was determined based on the measured exenatide content of the dried PLGA microspheres. The prepared suspensions were administered to mice at an exenatide dose of 4 mg/3 ml/kg via a single SC injection using a 23 or 25‐gauge needle, as reported previously with minor modifications.^55^, ^59^, ^101^, ^102^

On the first day after SC injection, blood samples were obtained from the orbital sinus using heparinized glass capillary tubes at pre‐dose and post‐dose 2, 8, and 24 h to evaluate in vivo IBR. From the second day after administration, blood samples were collected at 8 a.m. on days 3, 6, 9, 12, 17, 24, 28, and 32. All blood samples were collected in microcentrifuge tubes with EDTA as a coagulant, and immediately agitated by vortex mixing. After centrifugation (1600× g, 15 min, 4°C), the plasma proteins were removed from the separated supernatant by filtration using a SEP‐column (RK‐SEPCOL‐1, Phoenix Pharmaceuticals Inc., CA, USA). The plasma samples were stored at −80°C until further analysis.

The exenatide concentration in the collected plasma samples was measured using an exendin‐4 ELISA kit (EK‐070‐94CE Phoenix Pharmaceuticals Inc., CA, USA) following the manufacturer's protocol.^55^ A microplate photometer (Infinite F50; Tecan, Männedorf, Switzerland) was used to read the absorbance at 450 nm, and the concentration of exenatide was estimated by extrapolation based on a standard curve with good linearity. Direct determinations from the experimentally obtained PK data were used to determine the maximum plasma concentration (*C*_max_) and the time required to reach *C*_max_ (*T*_max_). In addition, PKSolver software was used for the noncompartmental PK analyses of plasma exenatide concentrations vs. the time profile, and the area under the curve (AUC) was obtained.^103^ All data are presented as mean ± *SD*.

#### 2.9.2 Pharmacodynamic study to evaluate therapeutic efficacy

Nonfasting glucose levels, food intake, and changes in body weight were measured to investigate in vivo efficacy as PD studies (*n* = 6–8). These PD studies for the ELPM_SE and ELPM_SFEE administration group were evaluated simultaneously with the PK study. Additional two groups administered with exenatide aqueous solution (140 μg/3 ml/kg, SC injection twice a day) and an aqueous suspension of blank PLGA microspheres (prepared using SE, single SC injection), respectively, were used as controls to be compared with two groups administered with exenatide‐loaded microsphere formulations prepared using SE and the optimized SFEE process. The exenatide aqueous solution and aqueous suspension of empty PLGA microspheres were prepared using the aqueous dispersion medium described above.

For the measurement of nonfasting blood glucose levels, 10 μl of blood samples collected at prescheduled time points were withdrawn using a micropipette and analyzed using a blood glucose meter (AccuCheck Active, Roche Diagnostics, Germany).

Food intake and changes in body weight were monitored thrice a week and once every 3 days, respectively. The average food intake and average body weight change per mouse were determined by dividing the total amount of food intake and total body weight in one cage by the number of animals per cage, respectively.

### 2.10 Statistical analysis

Significant differences were determined using an independent *t*‐test or one‐way ANOVA. All statistical analyses were performed using SPSS v12.0 software (IBM SPSS, Chicago, IL, USA).

## 3 RESULTS AND DISCUSSION

### 3.1 Evaluation of the fitted model and the effects of independent variables on each response

All experimental conditions, where each independent variable was combined using BBD, and the observed responses of particle size, SPAN value, EE, IBR, and residual solvent corresponding to each run are presented in Table 2. The statistical significance of the process parameters (used as independent variables) for the responses was investigated using ANOVA. In the following subsections, the mathematical model explaining the influence of process parameters on each response and their correlation will be discussed in detail.

The model *p*‐value, lack‐of‐fit, R^2^, R^2^ pred, R^2^ adj, and adep precision for the four applied mathematical fit models are given in Table 2. In addition, more detailed statistical analysis results including *F*‐value, coefficient estimates, and PRESS are presented in Table S1 (supplementary material). These values were used to check the degree of fit of the proposed models and the model predictive ability and to select the most adequate mathematical model under the experimental conditions.

Generally, R^2^ should be at least 0.5 for preferable fitting.^94^ In addition, a mathematical model with a better correlation between independent variables and the response has larger values of R^2^ and R^2^ adj.^104^, ^105^, ^106^ Regarding the PRESS value, the smaller the PRESS value, the higher the predictive power, which can prevent the chance of overfitting a mathematical relationship.

Based on the model analysis for all the responses in Table 2, the linear and quadratic models were found to have the most desirable R^2^, R^2^ adj, and R^2^ pred values for particle size and the other four responses (including SPAN value, EE, IBR, and residual solvent), respectively. This indicates that these models are adequate to fit well, with a proper goodness of fit for each corresponding response. Further, the relatively small PRESS value of each selected model indicates a good prediction of the experimental results when compared to that with other models.^107^ Consequently, the linear and quadratic models for particle size and the quadratic model for particle size and the other four responses were selected as the most suitable statistical models, *X*_1_: pressure (bar), *X*_2_: temperature (°C), *X*_3_: stirring rate (rpm), and *X*_4_: flow ratio by applying regression analysis to the experimental data. The mathematical relationships obtained in the polynomial equations in terms of the actual factors for all the responses are given in Table 2.

Typically, the derived positive or negative signs of the factors in the model equation indicate a positive or negative or synergistic or antagonistic effect on the responses for the factors.^108^, ^109^, ^110^

The ANOVA results (Table 2) of the selected model for each response indicate that these models are statistically significant, as evident from the low *p*‐values (<0.0001). The *F*‐values of the selected model for the particle size, SPAN value, EE, IBR, and residual solvent were 12.90, 16.92, 11.52, 28.69, and 34.22, respectively. Further, both models showed a statistically insignificant lack of fit, indicating that there was no significant difference between the model error and replicate error. The obtained adequate precision values of the fitted models were 11.44, 13.30, 12.07, 18.14, and 20.38 for particle size, SPAN value, EE, IBR, and residual solvent, respectively. The adequate precision measures the signal‐to‐noise ratio, and a ratio greater than 4 is generally desirable. This indicated that the selected model exhibited an adequate signal. Thus, these models can be used to navigate the design space for all responses.

#### 3.1.1 Particle size and its distribution

As described above, the SR kinetics of drugs from PLGA microspheres can be controlled by particle size and distribution. Thus, the desired drug release profile with uniformity can be obtained by optimizing the PSD. Microspheres that are “too small” exhibit poor SR efficiency; they may also migrate from the site of injection, resulting in undesirable drug release. in contrast, spheres that are “too large” may not easily pass through a syringe needle.^111^ Thus, particle size and SPAN value, which is an indicator of PSD, were selected as critical responses for quality control of the SR PLGA microspheres, and were obtained experimentally.^112^ From the experimental responses presented in Table 2, particle size and SPAN value were found to vary in the range of 21.31–31.5 μm and of 1.16–1.73, respectively.

The coefficient estimates and standardized main effect (SME) values for factors obtained from the polynomial equations of the selected models for particle size and SPAN values are presented in Table S1. The SME values were calculated by dividing the main effect by its standard error. A larger SME value indicate a more significant impact on the designated responses. The three independent variables, including temperature, stirring rate, and flow ratio, were found to be the major process parameters, which have significant effects on both the particle size and SPAN value with large linear coefficients and SME values, as well as small *p*‐values (<0.01) (Table S1). Further, four linear and quadratic terms for all four factors significantly affected the SPAN values (*p* < 0.01). The significance of these effects could be ordered as: for particle size, stirring rate > flow ratio > temperature, and for SPAN value, stirring rate > temperature > stirring rate^2^ > temperature^2^ > flow ratio > pressure > pressure^2^ > flow ratio^2^.

**FIGURE 2:**
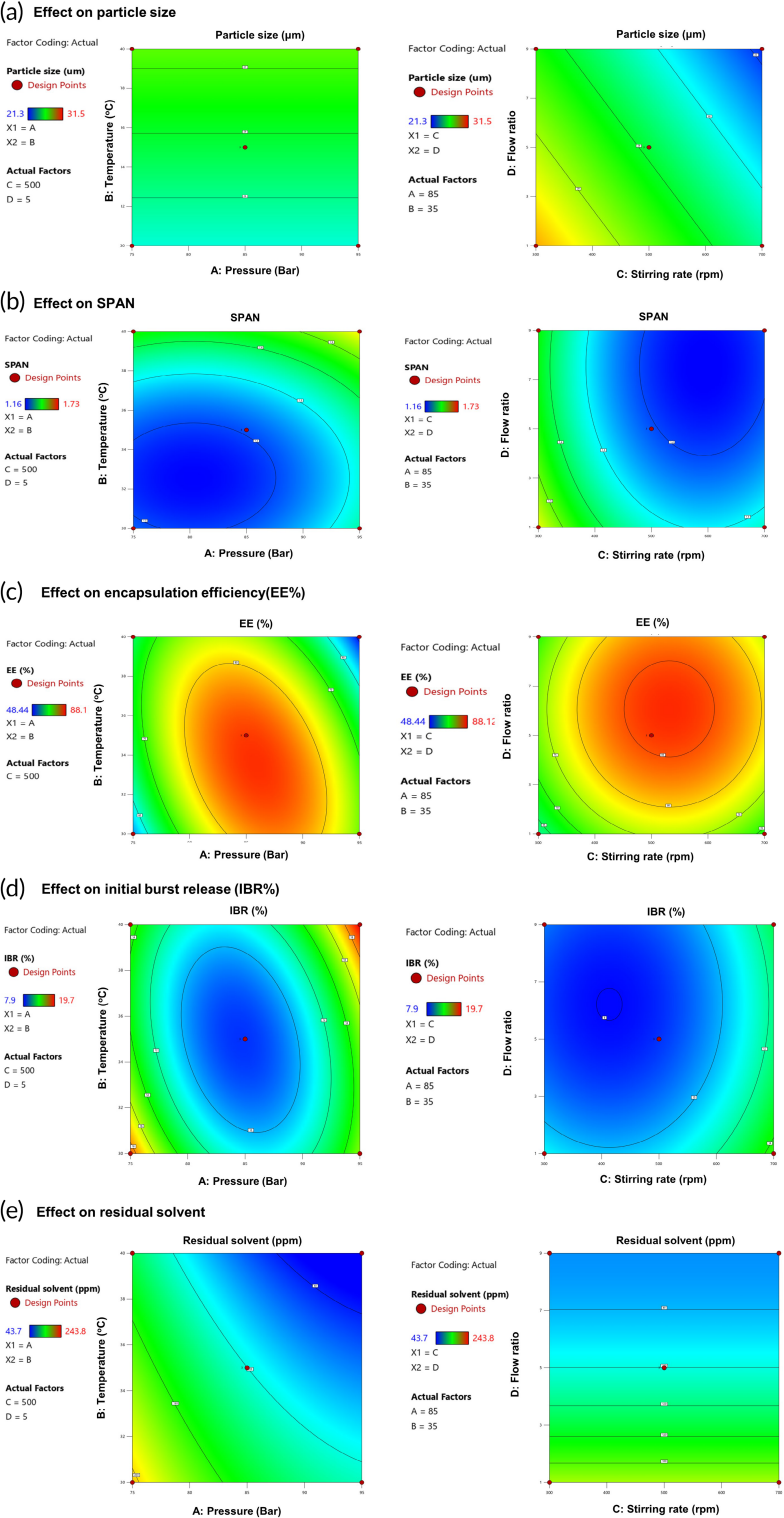
Counter plots showing the main effects of independent variables on responses

To visually evaluate the effects of independent variables on particle size and SPAN value, contour plots are shown in Figure 2a,b. From the plus or minus sign of the terms of actual factors for the response of particle size, it was confirmed that particle size increased as the temperature increased, whereas the particle size decreased as the stirring rate and flow ratio increased. Overall, for the SPAN value, there was a remarkable change in SPAN value with temperature, and the SPAN values decreased with an increase in all independent variables. In particular, it was clearly observed that the peak values in the counter plots of SPAN were within the experimental range, indicating a significant quadratic effect of the applied factors. Among the significant factors, temperature had the most remarkable quadratic effect, but the flow rate was relatively small. For the temperature variable, SPAN value decreased at temperatures close to 35°C, whereas SPAN value increased oppositely when the temperature increased above 35°C, indicating that an excessive increase in temperature might lead to a decrease in the uniformity of the PSD.^113^

In the SFEE process, the droplets of the oil phase undergo swelling after SC‐CO_2_ diffusion, and undergo shrinking due to DCM diffusion out of the drop into the external water phase.^1^, ^16^, ^114^ In contrast, the SC‐CO_2_ concentration in the drop could be relatively increased. The emulsion drop behaved as a miniature gas antisolvent precipitator, and the PLGA polymer was fabricated as a microsphere. During this fabrication process, the higher temperature and pressure decreased the shrinkage of the DCM drop, resulting in an increase in the particle size. In particular, the different thermodynamic behaviors in the emulsion could be attributed to the changes in temperature and pressure during the SFEE process, resulting in a difference in the particle size of the microspheres prepared by the SFEE process.

**FIGURE 3:**
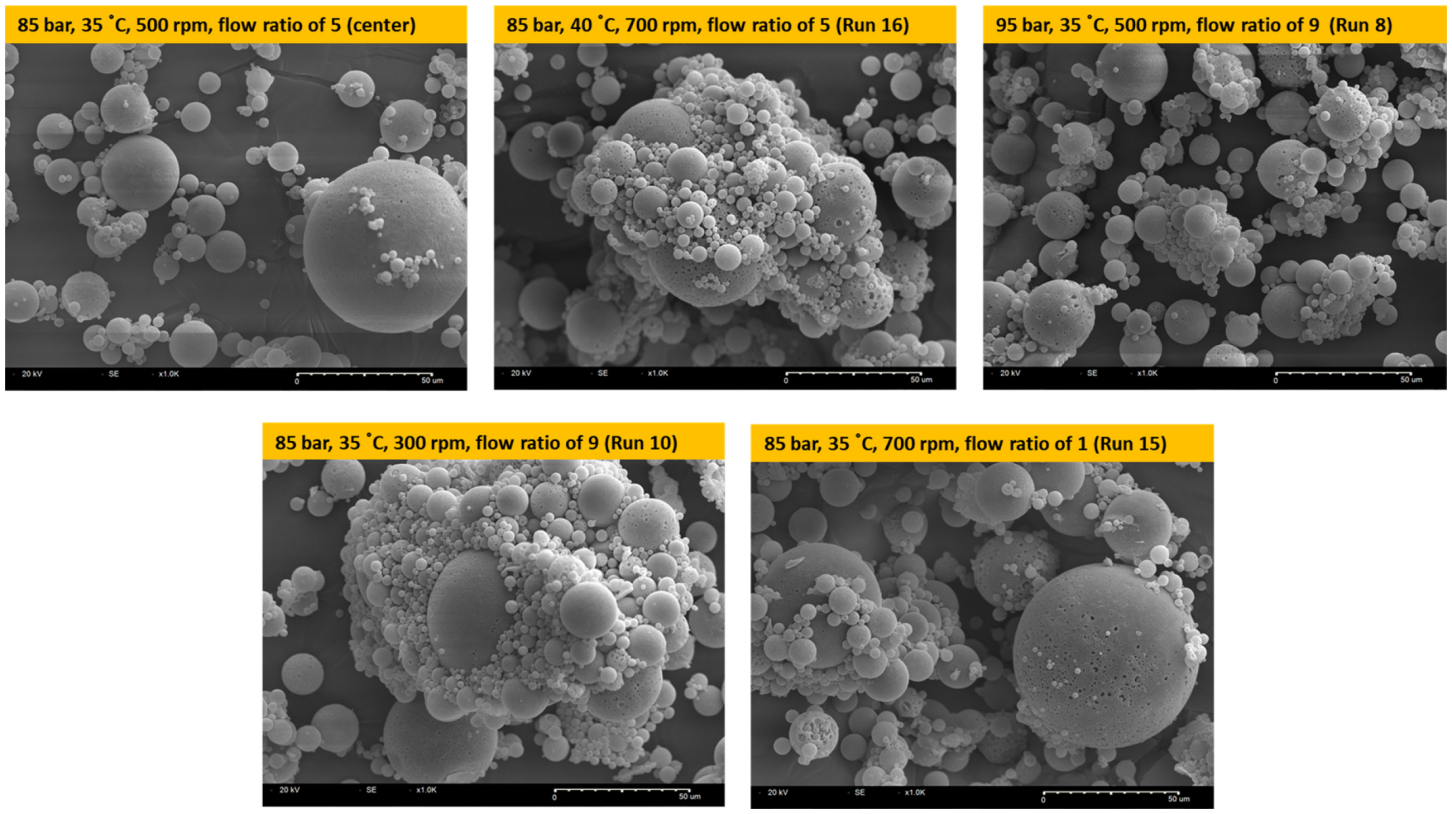
Scanning electron micrographs (SEM) of exenatide‐loaded PLGA microspheres prepared using supercritical fluid extraction of emulsions (SFEE)

As described above, the particle size, in terms of VMD, of the microspheres increased significantly with increasing temperature. This result can be explained by the fact that the shrinking rate of the DCM droplet slows down at higher temperatures because at such high temperatures exceeding the boiling point of DCM at high pressure, diffusion of SC‐CO_2_ into the DCM droplet and diffusion of DCM into the outer aqueous phase would be very rapid. Thus, excessive expansion of the DCM drop occurs at high temperatures, and fabrication of PLGA occurs too quickly before complete drop shrinkage caused by the high DCM removal efficiency via the combined effect of extraction and evaporation, leading to a larger particle size of the obtained PLGA microspheres. This decreased shrinking rate and excessively increased evaporation of DCM can lead to a decrease in the density and more porous internal structure of microspheres (Figure 3). In addition, the occurrence of particle agglomeration due to an increase in temperature, decrease in stirring rate or flow ratio, which will be discussed later in this subsection, can also be another major reason of the increase in particle size.

However, the insignificant effect of pressure on particle size was unexpectedly observed, and it can be explained as follows. At low pressure, the volume of the drop first increased until it reached a maximum value, and then decreased until a dense thin layer of polymer was fabricated. Mattea et al.^114^ reported a sequence of images for a typical experiment of DCM drop evolution in water during the SFE procedure: at relatively low pressure, (a) the initial state of the drop before CO_2_ was introduced into the extraction cell, (b, c) the change in the refractive index (RI) and the point where the RI of the DCM drop and water phase became equal, (d) the maximum volume point, and (e) the volume reduction and fabrication of the PLGA polymer. On the contrary, during SFE carried out at high pressure above 100 bar, the volume of the DCM drop increased continuously until a dense thin layer of polymer was fabricated: (a) the initial stage of the DCM drop, (b) the point where the RI of the drop and surrounding media became equal, (c) increase in the drop volume, and (d) the formation of a dense thin layer of polymer. Thus, the drop volume increased continuously until a thin dense layer of PLGA was formed. Further, as the SFEE process progressed, the CO_2_ concentration in the drop increased, and the equilibrium constraints tended to slow CO_2_ diffusion into the emulsion droplet until the volume variation became negative (shrinking rate was positive) by comparatively faster diffusion out of DCM from the emulsion droplet. Based on these theories, it was estimated that a significant increase in particle size would not be observed with an increase in pressure within the pressure range used in this study, because the pressure conditions were all less than 100 bar.

On the other hand, it could also be supposed that if the diffusion out of DCM was accelerated by a combination of other factors at a relatively high pressure (not more than 100 bar), the particle size might be decreased with an increase in pressure. However, in this study, pressure had no significant effect on the particle size of the microspheres prepared using the SFEE process. This result may be attributed to that CO_2_ diffusion was not limited by equilibrium conditions at 75–95 bar in all experimental runs of this study; thus, an increase in pressure cannot significantly enhance drop shrinkage due to DCM diffusion from the emulsion droplet predominantly more than that of drop swelling because of DCM diffusion into the emulsion droplet.

As stated, the influence of the four process variables on the SPAN value is significant. The increase in SPAN values above 35°C and 85 bar was attributed to particle agglomeration in the suspension, even though the presence of an outer aqueous phase may prevent PLGA microsphere coalescence.^1^ The fact that SC‐CO_2_ can produce “swelling” and “*T*_g_ lowering” effects on PLGA is well known based on literature, confirming that the PLGA microsphere could be quite unstable in the presence of SC‐CO_2_.^1^, ^115^, ^116^, ^117^, ^118^ Further, it is already reported that PLGA glass transition can occur at 40°C in SC‐CO_2_. As shown in Figure 3, the SEM image analysis confirmed that the PLGA microspheres prepared at temperatures above 35°C and 85 bar show particle agglomeration. There is another suggested reason for microsphere agglomeration in the suspension at conditions above 35°C and 85 bar. The relationship between pressure and interfacial tension has been well studied by other researchers. The volume of CO_2_ in the drop initially increased intensely, and could be considered to be linearly related to time. Furthermore, the interfacial tension between the drop and aqueous phase increased during the SFEE process, thereby destabilizing the emulsion. This instability can cause coalescence and agglomeration of PLGA microspheres.^114^ Therefore, excessive temperature and pressure should be avoided during the SFEE process for preparing PLGA microspheres.

Further, the SPAN value also increased as the SFEE process moved away from 35°C and 85 bar. Different behaviors of CO_2_ and volatile organic solvents have been observed more clearly below and above the critical point of CO_2_. CO_2_ can be mixed more homogenously with organic solvents in the supercritical state, and can extract organic solvents more efficiently. However, process conditions close to 30°C and 75 bar are not supercritical conditions above the critical point of CO_2_ (critical temperature 31°C and critical pressure 74 bar). Thus, in the experiments performed at 75 bar, the diffusion of CO_2_ to the DCM drop could be limited by the relatively low equilibrium concentration of DCM in CO_2_ as well as the lower solubility of CO_2_ in water, which can reduce the extraction efficiency of DCM through the outer water phase in W/O/W emulsions. In this case, the longer the organic solvent stays on the microsphere surface, the chances of contact between particles are increased, which can lead to more agglomeration of PLGA microspheres via easier fusion of particle surfaces in the presence of the solvent.

The particle size and SPAN value increased as the stirring rate or flow ratio decreased under overall temperature and pressure conditions. The increased particle size and SPAN value of microspheres prepared at a low stirring rate or low flow ratio were strongly assumed to be caused by the coalescence of emulsion droplets due to the low mixing efficiency and slow formation of a dense thin layer of PLGA. The result of this tendency of particle change by stirring rate and flow ratio was also observed in SEM (Figure 3).

#### 3.1.2 EE and IBR

The experimentally obtained EE and IBR values varied in the range of 50.40%–88.12% and 7.90%–19.70%, respectively (Table 2).

All linear terms and four quadratic terms corresponding to all factors, including pressure, temperature, stirring rate, and flow ratio, were determined as major process parameters with significant effects on EE and IBR, having large coefficients and SME values in terms of coded factors, as well as small *p*‐values (<0.01) (Tables 2 and S1). Further, there was a significant interaction effect between pressure and temperature (*p* < 0.01), and the positive sign of this interaction term corresponding to actual factors indicates a synergistic effect on EE and IBR. The order of significance for these effects, determined based on the SME value, was as follows: for EE, pressure^2^ > stirring rate^2^ > pressure· temperature > temperature^2^ > flow ratio^2^ > flow ratio > temperature > stirring rate > pressure, and for IBR, pressure^2^ > stirring rate > temperature^2^ > stirring rate^2^ > pressure· temperature > flow ratio^2^ > flow ratio > pressure > temperature.

The contour plots in Figure 2c,d show the effects of the independent variables on the EE and IBR of ELPMs prepared using the SFEE process. The clearly observed peak values in the counter plots for EE and IBR indicate the significant quadratic effects of pressure, temperature, stirring rate, and flow ratio as the major process parameters. The factor that showed the most pronounced effect on EE was the quadratic term of pressure, and the linear term corresponding to stirring rate showed the most significant effect on IBR.

Interestingly, Figure 2c,d also shows that overall, EE decreased and IBR increased as the process conditions moved away from the point corresponding to the peak value. The estimated process condition corresponding to the peak value was close to the center point at 35°C, 85 bar, 500 rpm stirring rate, and a flow ratio of 5.

At low temperature, pressure, stirring rate, and flow ratio, because the extraction efficiency is lowered, the fabrication of the outer shell is delayed or a less dense outer layer of the PLGA microspheres is formed, and the EE is lowered because the inner drug can be lost and diffused into the outer phase during this time. However, if the temperature, pressure, stirring rate, and flow ratio are excessively increased, the diffusion rate of the inner drug can increase, and thus the EE tends to decrease because the drug can escape from the inner aqueous phase to the outer aqueous phase via higher mass transfer of the encapsulated drug.^119^, ^120^, ^121^ Further, the porosity of the fabricated polymer can increase owing to the formation of channels for the mass transfer of the internal phase to the outer phase.^122^, ^123^, ^124^, ^125^, ^126^

It was unexpected that the increase in pressure and temperature above 35°C and 85 bar caused a decrease in EE (Figure 2c). The drug EE was expected to be high at higher temperatures and pressures because microspheres prepared under such conditions solidify rapidly, forming a dense thin outer layer of microspheres caused by the high efficiency of diffusion and extraction rate of SC‐CO_2_ at high temperatures and pressures. Naturally, a dense outer layer provides impedance, hindering peptide diffusion through the polymer layer during the solvent removal process. However, EE decreased as the temperature and pressure increased above 35°C and 85 bar, respectively. The preparation temperature and pressure during the SFEE process could affect the mass transfer rate of SC‐CO_2_ and DCM, as well as the mass transfer and solubility of the peptide. Although faster formation of the dense outer layer may reduce peptide loss, an increase in peptide solubility at higher temperatures and faster mass transfer may also increase the amount of exenatide leaving the dispersed phase during microsphere formation.^127^, ^128^, ^129^ Peptide denaturation at high temperatures may also attribute to the decreased EE owing to degradation of the encapsulated peptide.^130^, ^131^, ^132^

In addition, the high pressure inside the hydrated PLGA microspheres caused by the combination of high temperature and pressure during SFEE process can induce microsphere breakage. This negative effect could be increased at a temperature higher than the *T*_g_ of PLGA because the dense outer layer of the microspheres became more relaxable owing to the glass transition of the PLGA polymer. Further, thermodynamic equilibrium exists between the CO_2_ dissolved in the aqueous phase and that dissolved in the polymer particles after complete precipitation of PLGA during the SFEE process. Typically, it was reported that between 10% and 30% (w/w%) of CO_2_ can be dissolved in the PLGA polymer, depending on the process pressure and temperature. The dissolved SC‐CO_2_ in the polymer has an effect of “*T*_g_ lowering” on polymer like PLGA, by which this peptide loss can be increased.^1^ The decrease in *T*_g_ also can be influenced by the processing temperature depending on the characteristics and molecular weight of the polymer, resulting in the formation of CO_2_ plasticized polymer particles.^115^ This plasticization of the polymer can be beneficial for facilitating faster solvent removal from the polymer phase, but not for forming a hard and rigid polymer layer.

Overall, the IBR value was found to change in the direction opposite to that of the EE for all four process parameters.^133^ In general, the IBR of the drug observed in microspheres is probably attributed to poorly entrapped peptides and peptides loosely attached to the internal and outer surfaces.^4^ Further, the high porosity of the fabricated polymer, which is strongly correlated with a low EE, may also cause an increase in IBR.^134^ Thus, when the EE of the prepared microspheres is low, more drugs are present on the external surface of the microspheres, and can be adsorbed onto the microsphere surface, resulting in a larger IBR.^135^, ^136^, ^137^

#### 3.1.3 Residual solvent on PLGA microspheres

The experimentally obtained residual solvent for all runs varied in the range of 43.7–243.8 ppm (Table 2).

Three linear terms corresponding to pressure, temperature, and flow ratio and two quadratic terms corresponding to pressure and flow ratio had significant effects on the residual solvent with large coefficients and SME values in terms of coded factors, as well as small *p*‐values (<0.01). Based on this result, we confirmed that the pressure, temperature, and flow ratio are the major process parameters for residual solvents. The order of significance for these effects, determined based on the SME value, was as follows: flow ratio > pressure > temperature > flow ratio^2^ > temperature^2^.

The contour plots in Figure 2e show the effects of the process parameters on the residual solvent of ELPMs prepared using the SFEE process. Among significant factors, the linear terms of pressure, temperature, and flow ratio had remarkable effects on the residual solvent, but the effects of quadratic terms corresponding to the pressure and flow ratio were relatively small. Moreover, as shown in the contour plots (Figure 2e), PLGA microspheres with a lower residual solvent content were obtained as the temperature, pressure, and flow ratio increased. This result was attributed to the higher mixing efficiency of CO_2_ and DCM, and the subsequently improved DCM extraction efficiency.^34^

### 3.2 Determining a desirable range for the combination of various variables in the SFEE process by graphical optimization

Based on the above experimental results within the design space applied in this study, optimization was carried out to verify the fitted model after establishing polynomial equations to describe the relationship between the factors and the processed responses by setting the acceptable target ranges of responses, as given in Table 1.

The target range of particle size was set to 20–30 μm because it was reported that the mean particle size of Bydureon™ microspheres is approximately 50 μm, which requires the use of painfully large, 23‐gauge needles for SC injection, thus decreasing patient compliance.^56^, ^57^, ^58^ The SR exenatide delivery system including Bydureon™ or Byetta® Long‐Acting Release (LAR) was developed by Amylin Pharmaceuticals/Eli Lilly using Alkermes's water‐in‐oil‐in‐oil (W/O/O) coacervation technology.^55^ Based on the results of our previous and preliminary studies, the target range of the SPAN value was selected as 1.3 or less. The target range of EE was set at a minimum of 85%, which could be easily achieved through SFEE, with an ideal of 100% as the maximum. Based on the results of our previous studies and the in vivo evaluation reports on the IBR of the exenatide long‐acting formulation, the target range of the IBR was set to a minimum of 3.3% and a maximum of 10%, assuming a SR formulation for 30 days. The ICH guidelines stipulate that DCM be less than 600 ppm, but to apply a stricter management standard, 150 ppm, 1/4th of the standard set by ICH guideline, was set as the maximum target range.^58^

**FIGURE 4:**
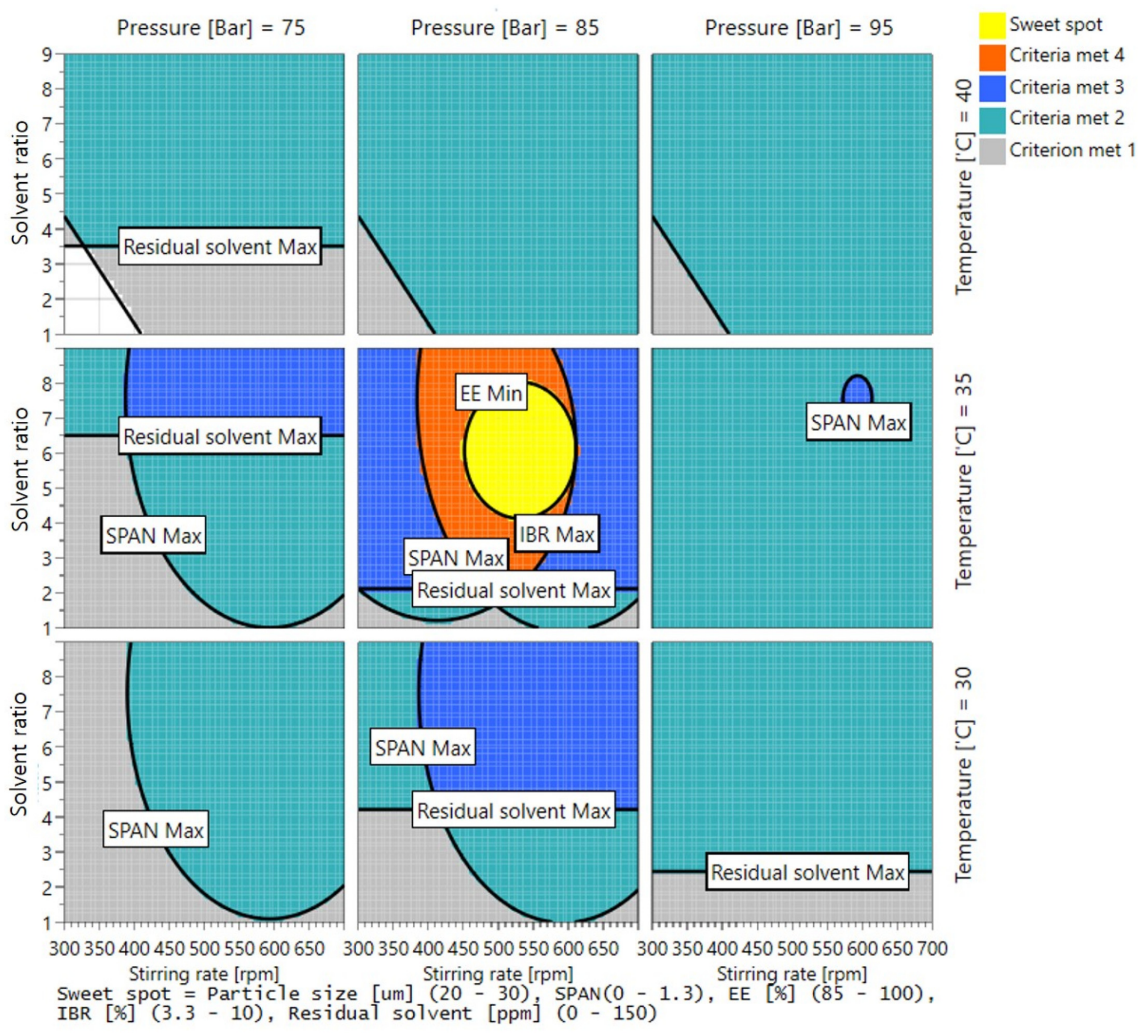
Four‐dimensional sweet‐spot plots obtained by plotting the factors (*X*1–*X*4) leading to optimal responses

The 4D sweet‐spot plots shown in Figure 4 were obtained using the four factors and an acceptable range of responses, as given in Table 1. The model error and process uncertainty were not considered because a deterministic regression model was applied for the construction of this sweet‐spot plot. The yellow area is the sweet spot, which clearly shows the range of conditions for combinations of different process parameters that meet the criteria for all responses. The condition associated with the center point was thus confirmed to be suitable, as it was located within the yellow area covering all target response ranges. Thus, the ELPMs prepared using the SFEE process at 35°C, 85 bar, 500 rpm stirring rate, and a flow rate of 5, were then used for evaluating the following results.

As such, a fundamental understanding of the effects of the SFEE process parameters on the pharmaceutical properties of ELPM and information on the range of suitable process conditions that can derive the desirable pharmaceutical quality of SR formulation can be applied to commercially scale‐up manufacturing of PLGA microsphere as translational research for successful commercial production and clinical use.

### 3.3 Comparison of PLGA microspheres prepared using SFEE and solvent evaporation

**FIGURE 5:**
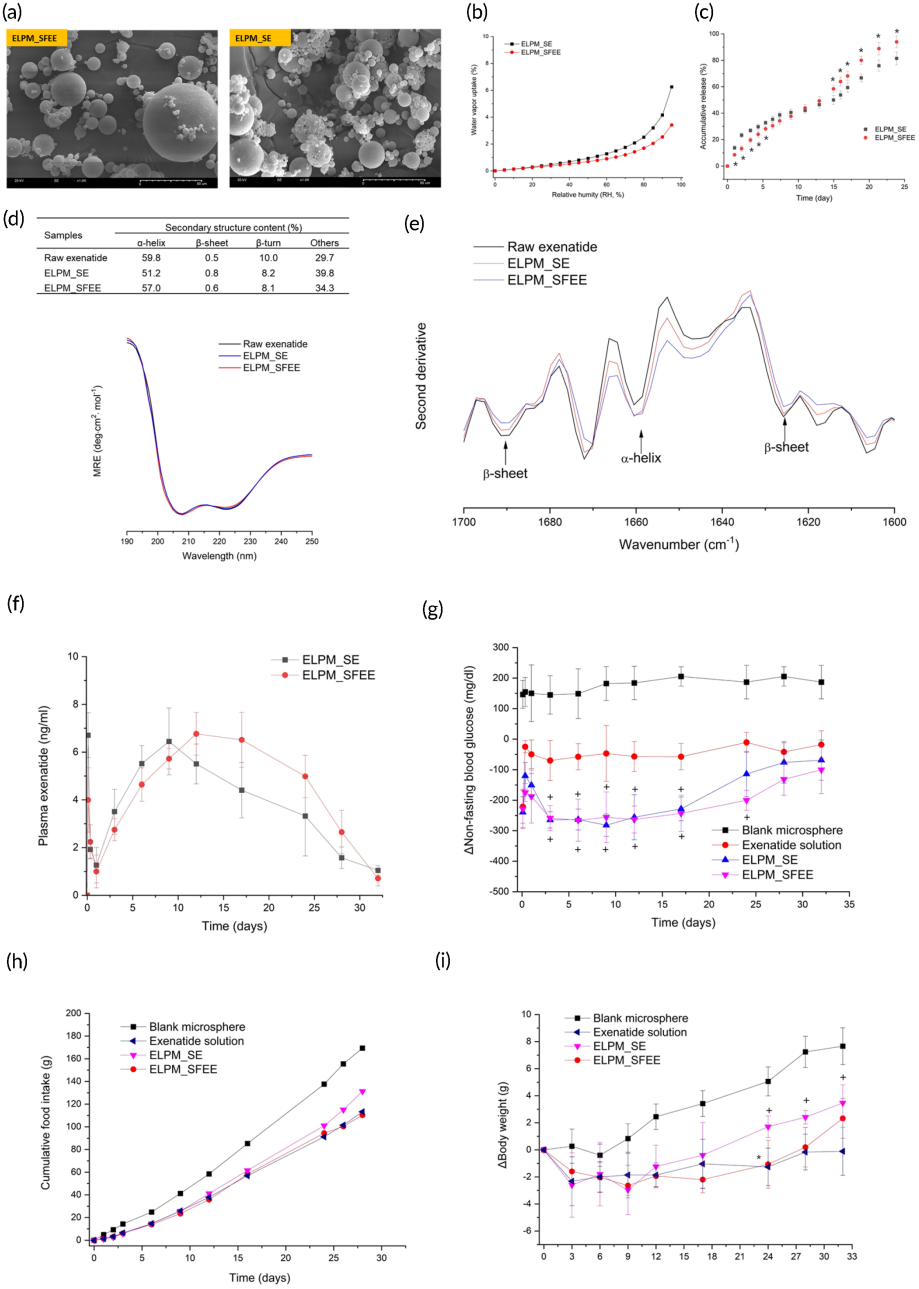
Comparison of (a) scanning electron micrographs (SEM), (b) water vapor sorption profiles over increasing relative humidity (RH%), and (c) in vitro exenatide release (*n* = 3, pH 7.4 PBS, 37°C) between exenatide loaded PLGA microsphere prepared by SE (ELPM_SE) and SFE process (ELPM_SFEE, (d) circular dichroism spectra with change in the conformation type as determined using the Bestsel secondary structure analysis tool and (e) second derivative Fourier‐transform infrared (FT‐IR) spectra for comparison of secondary structure between raw exenatide and extracted exenatide from ELPM_SE and ELPM_SFEE, (f) pharmacokinetic profiles and (g) changes in nonfasting blood glucose levels, (h) food intake, and (i) weight gain in streptozotocin‐induced diabetic mice: a single SC injection with ELPM_SE or ELPM_SFEE, twice daily SC injection with exenatide solution, or a single SC injection with blank PLGA microsphere. (“*” indicates significant difference (*P* < 0.05) compared to ELPM_SE, “+” indicates significant difference (*P* < 0.05) compared to exenatide solution).

As mentioned above, PLGA microspheres obtained from the center point of the design space that satisfy all response criteria were compared with PLGA microspheres prepared using a previously reported SE method through various solid‐state characterizations and in vitro and in vivo evaluations (Table 3 and Figure 5).

**TABLE 3:** In vitro characteristics and in vivo pharmacokinetic parameters of exenatide following a single SC injection to streptozotocin‐induced diabetic mice of ELPM_SE and ELPM_SFEE.

In vitro characterization (*n* = 3)	Group	
	ELPM_SE	ELPM_SFEE
Particle size (VMD, um)	28.4 ± 2.7	23.2 ± 1.8*
SPAN^a^	1.62 ± 0.07	1.21 ± 0.03*
Residual solvent (ppm)	627.5 ± 73.8	85.4 ± 6.1*
Surface area (m^2^/g)	1.72 ± 0.12	1.21 ± 0.09
Pore size (nm)	4.2 ± 0.3	3.1 ± 0.2
EE (%)	73.6 ± 2.7	82.9 ± 2.1
IBR^b^ (%)	13.9 ± 1.4	8.5 ± 0.7
*R*_max_ ^c^ (%)	81.4 ± 4.8	93.9 ± 4.4

In vivo pharmacokinetic		Group	
Period	Parameters	ELPM_SE	ELPM_SFEE
Before 24 h	*T*_max_0–24 h_ (hours)	2.0 ± 0.0	2.0 ± 0.0
	C_max_0–24 h_ (ng/ml)	6.7 ± 0.94	3.99 ± 1.35*
From 24 h	*T*_max_1–32 days_ (days)	8.0 ± 1.7	13.7 ± 2.9*
	*C*_max_1–32 days_ (ng/ml)	6.47 ± 1.37	6.97 ± 0.70
	AUC__0–32 days_ (ng day/ml)	123.4 ± 28.8	146.6 ± 16.6

*Note*: Data are presented as the means ± standard deviation (*SD*).

Abbreviations: EE, encapsulation efficiency; SPEE, supercritical fluid extraction of emulsions; VMD, volume mean diameter.

^a^ Calculated as the ratio of (D90%–D10%) to D50%, where DN% indicates the volume particle diameter at each cumulative volume percentage.

^b^ In vitro initial burst release (IBR) measured on the first day.

^c^ In vitro maximum accumulated drug release % for 24 days.

* Statistically significant difference compared to ELPM_SE (*p* < 0.05).

#### 3.3.1 PSD and morphology

The particle size of the ELPMs prepared using the SFEE process (ELPM_SFEE) was smaller than that prepared using the SE process (ELPM_SE) (Table 3). This may be attributed to different extraction mechanisms with volume expansion and shrinking rates during extraction. In addition, the smaller SPAN values of ELPM_SFEE than ELPM_SE shows a more uniform PSD (Table 3). The SEM images in Figure 5a indicated that the morphology and surface properties of ELPM_SFEE were more spherical and smoother than those prepared using SE. Many previous studies have reported that polydispersed PLGA microspheres can be generated using conventional microsphere preparation techniques such as SE, and must be filtered or sieved to isolate particles within the desired particle size range.^3^ This result may be attributed to the higher extraction efficiency of the SFEE process than that of the SE process. In particular, the fast solidification of the dense outer PLGA layer using the SFEE process may prevent the easy release of the inner water phase during the extraction process, allowing the preparation of a spherical microsphere with a smooth surface.^16^

Moreover, the mean particle size of ELPM_SFEE was approximately 20 μm, which was much smaller than those of ELPM_SE and Byetta® LAR microspheres (i.e., approximately 50 μm). Byetta® LAR requires the use of painfully large, 23‐gauge needles for SC injection and, thus, decreases patient compliance.^59^ In contrast, an injectability test confirmed that only the suspension of ELPM_SFEE for SC injection can be injected through a 25‐gauge needle. Thus, ELPM_SFEE with a smaller particle size could improve patient compliance.

#### 3.3.2 Residual organic solvent

Comparatively, the extraction of DCM from the same volume of emulsion using the SE process at 35°C required approximately 7 h to reach DCM levels below 600 ppm. In contrast, with the SFEE method, 5 h was sufficient for the level of residual DCM to fall below 150 ppm (Table 3). As mentioned above, these results are attributed to the higher mixing efficiency of CO_2_ and DCM, and the subsequent improved DCM extraction efficiency in the SFEE process than that in the SE process. Therefore, from these residual solvent analysis results, it was confirmed that the SFEE technology is not only a production technology for SR microspheres that can efficiently minimize the presence of organic solvents in a short process time, but also have the potential clinical benefits which can prevent the increase in the risk of toxicity in patients caused by residual organic solvents.

#### 3.3.3 BET specific surface area and water vapor sorption

The blank PLGA microspheres without exenatide were prepared under the optimized SFEE process conditions as described above (at center point), and used as a substitute for material sparing in BET measurements requiring large amounts of sample. Blank microspheres were also prepared using SE for comparison. In both processes, the presence or absence of exenatide did not significantly affect the particle size of the microspheres.

As presented in Table 3, the surface area and pore size of blank microsphere prepared by the SFEE process were significantly smaller than those prepared using the SE process. This result was also confirmed by the water vapor sorption results (Figure 5b) indicating that the degree of water vapor sorption of ELPM_SFEE can be significantly lower than that of ELPM_SE. The water channels connecting the internal and outer water phases can be formed during PLGA fabrication using both evaporation and extraction processes resulting in pores in the microspheres. However, in the SE process, these channels in PLGA microspheres can be formed excessively owing to the generation of vaporized gas. This difference is presumed to have led to the observed results. This also indicates that the stability of ELPMs prepared using the SFEE process during storage could be improved by lowering the decomposition rate of peptides owing to the low moisture content. These results also support the PSD and morphology results described above. In particular, some small pores were rarely observed in the SEM images of ELPM_SE but none were observed for ELPM_SFEE.

#### 3.3.4 EE, IBR, and sustained release profile

The EE of the SFEE microspheres containing exenatide was higher than that of SE microspheres (Table 3) because PLGA microspheres prepared using the SFEE process solidify rapidly, forming a dense thin outer layer of microspheres due to the high efficiency of diffusion and extraction rate of SC‐CO_2_ when compared with the SE process. These results indicate that the outer layer of PLGA microspheres prepared using the SFEE process was denser and thicker than that of the SE‐processed PLGA microspheres. Naturally, a dense outer layer provides impedance, hindering peptide diffusion through the polymer layer during solvent removal.^138^, ^139^, ^140^

Figure 5c shows the cumulative release of exenatide from ELPM in vitro. A typical triphasic release pattern was observed for both ELPM_SE and ELPM_SFEE, with IBR followed by a more controlled secondary phase and accelerated drug release in the third phase.^54^ As expected, the IBR of the SFEE microspheres with higher EE values was lower than that of the SE microspheres (Table 3).

Low EE and high IBR of ELPM_SE mean that exenatide molecules exiting the outer water phase leaked from inner water phase through the water channels are more adsorbed on the surface of ELPM_SE than ELPM_SFEE. Excessive formation of these water channels could be attributed to the larger pore size of the PLGA microspheres prepared using SE, compared to SFEE, hence resulting in the increased IBR of ELPM_SE by the larger surface area due to the pores of microsphere.^141^ In addition, a polymer impregnated with CO_2_ at a higher pressure, within a certain range of pressure and temperature, has been reported to show relatively low porosity in many research cases.^138^, ^142^, ^143^, ^144^ Based on this hypothesis, the BET specific surface area (Table 3) and water vapor sorption results (Figure 5b) can explain why the EE and IBR of the microspheres prepared using this process were smaller than those with the SE process.

The amount of exenatide released from ELPM_SE was less than 10% between day 7 and 14 in the secondary phase, which indicate it has obvious lag time, which means release of a drug that is inappropriately slow (Figure 5c). In addition, the final accumulated release percentage after 24 days (*R*_max_) was 81.4% for ELPM_SE, indicating relatively incomplete release of exenatide. On the other hand, ELPM_SFEE showed another preferred advantage of a reduced lag time during drug release in the secondary phase, compared with that of ELPM_SE. After the secondary release phase for ELPM_SFEE, exenatide was released sustainably for 24 days, and the final accumulated release percentage after 24 days was 93.9%, implying the possibility of optimal exenatide release in vivo. Overall, the cumulative amount of exenatide released from PLGA microspheres prepared using the SFEE process was higher than that from those produced using the SE process.

The peptide existing in the internal water phase of the double emulsion could be strongly interacted with PLGA fabricated at the W/O interface during the PLGA microsphere preparation process, hence inhibiting the complete release of peptide.^77^ Moreover, the accelerated drug release in the third phase can be caused by a dramatic change in the conditions for mass transport processes, when the drug is still present in its original state without aggregation and/or destabilization by interaction with PLGA.^4^ Thus, it is suggested that relatively more severe lag time upon drug release of ELPM_SE is due to that the undesirable interactions between exenatide and PLGA, including nonspecific adsorption and/or ionic interactions, may more exist in ELPM_SE than ELPM_SFEE.^145^

Consequently, the SFEE process was revealed to produce ELPMs with a more desirable SR property than that of PLGA microspheres produced using the commercially used conventional SE process.

#### 3.3.5 Secondary structure of exenatide in PLGA microspheres

Circular dichroism spectroscopy was used to evaluate the secondary structure of exenatide in aqueous solutions. Exenatide in the fabricated microspheres was extracted and used for CD analysis along with raw exenatide. As reported in our previous studies, the CD spectra of exenatide had two specific minima at wavelengths of 208 and 222 nm, indicating the presence of an α‐helix (Figure 5d).^71^, ^74^ The obtained CD spectroscopic data were also analyzed using the BeStSel analysis tool to determine secondary structures, such as α‐helices, β‐sheets, β‐turns, and other structures.^146^ Further, second‐derivative FT‐IR spectra were obtained for unprocessed exenatide and exenatide in microspheres prepared using the SE and SFEE processes (Figure 5e) to check for differences in secondary structure between samples. Intermolecular β‐sheets could be identified by the two peaks at 1692 and 1629 cm^−1^, and the peak assigned to the α‐helix could be observed at 1660 cm^−1^ in the amide I region.^66^, ^76^, ^97^ Typically, meaningful information about the peptide secondary structure could be obtained from these representative peaks in the second derivative FT‐IR spectra. There was no significant difference between the extracted exenatide from PLGA microspheres prepared using the SFEE and SE processes when compared to the unprocessed exenatide, indicating that the exenatide secondary structure in SE‐ and SFEE‐processed microspheres can be maintained without structural destabilization even after the completion of all manufacturing processes.^69^, ^77^ These results are presumed to be attributed to the stabilization of exenatide by the formulation containing various stabilizers and additives, which can ensure structural stability, as found in our previous study.

### 3.4 In vivo animal study in STZ‐induced diabetic mice

#### 3.4.1 PK profiles

The comparative PK profiles of ELPMs prepared using SE (ELPM_SE) and SFEE (ELPM_SFEE) and blank PLGA microspheres after a single SC injection are shown in Figure 5f. The calculated PK parameters are given in Table 3.

The curve in the PK profiles shows double peaks, and not a single peak, for both microspheres, typically indicating the IBR and subsequent SR of exenatide from PLGA microspheres.^55^ Specifically, the blood concentration of exenatide rapidly increased and immediately decreased within the first day after SC administration, which was observed in both ELPM_SE and ELPM_SFEE. Further, there was a clear difference in the degree of IBR between the two formulations. The in vivo IBR of exenatide is suggested to lead to the appearance of the first peak of exenatide plasma concentration in the PK profile.^74^ As presented in Table 3, the *C*_max_0–24 h_ obtained within the first day after SC injection were significantly lower (*p* < 0.05), for ELPM_SFEE than for ELPM_SE, indicating that ELPM_SFEE has a lower in vivo IBR than that of ELPM_SE.

After the first day, a gradual increase and substantial sustainment for 32 days were observed in the PK profile. *T*_max_1–32 days_ was significantly slower for ELPM_SFEE than for ELPM_SE (*p* < 0.05). These results show that ELPM_SFEE has better SR properties than those of ELPM_SE. Further, ELPM_SFEE showed slightly larger *C*_max_1–32 days_ and AUC__0–32 days_ than those of ELPM_SE, but the difference was not statistically significant (*p* > 0.05).

These PK results were consistent with the results of the in vitro experiments. The PK results showed that the release of exenatide from ELPM_SFEE was well‐sustained in STZ‐induced diabetic mice with low IBR and had an improved bioavailability when compared with that of ELPM_SE. Thus, the PK results indicate a more favorable in vivo SR property for ELPM_SFEE than for ELPM_SE. Further, ELPM_SFEE may have an improved in vivo sustained PK profile compared with that of Byetta® LAR, a commercial ELPM product, based on a report that it has poor SR characteristics with low release between days 4 and 17 in animal PK study.^55^ Like this, it has been reported that Byetta® LAR, comprising SR exenatide PLGA microspheres, has some problems. In particular, the weekly dose of exenatide in Byetta® LAR microspheres (i.e., single injection of 2 mg/human) is much higher (14–28 fold) than that of Byetta® (i.e., twice‐daily injection of 70–140 ug/human). The improper release of exenatide from Byetta® LAR in vivo may explain its poor pharmacokinetics and the necessity for a much higher dose.^60^ Another study reported that the drug burst release was high (approximately 45%) when they prepared microspheres following the commercialized ELPM formulation.^59^ These problems can increase the risk of adverse effects due to overdose of exenatide.^54^ From these results, it was shown that the ELPM prepared with SFEE may have better drug release properties compared to the existing commercialized ELPM formulation. This could indicate the clinical meaning that, based on the fundamental understanding of the SFEE process obtained in this study, if it can be successfully applied to the commercial production technology of SR microsphere, more improved ELPM preparations may be provided to patients in clinical practice.

#### 3.4.2 Pharmacodynamic therapeutic efficacy

The measured nonfasting glucose levels of the ELPM_SE, ELPM_SFEE, and blank PLGA microspheres after a single SC injection and twice daily SC injection of exenatide solution are shown in Figure 5g. For the empty PLGA microsphere‐injected group, nonfasting glucose concentrations increased progressively over 32 days, indicating no blood glucose‐lowering effect in STZ‐induced diabetic mice. Twice daily SC administration of exenatide solution had a slightly higher hypoglycemic efficacy compared with that of empty microsphere administration. In contrast, significantly reduced nonfasting glucose levels were observed in the two groups administered a single SC injection of ELPM_SE and ELPM_SFEE when compared with the twice daily SC injection of exenatide solution. In particular, ELPM_SFEE lowered nonfasting blood glucose concentrations more effectively than ELPM_SE, and this difference in hypoglycemic efficacy was significant. Overall, ELPM_SFEE, which has a lower IBR, higher EE, and sustained in vitro and in vivo release, showed proper in vivo therapeutic efficacy for hypoglycemic behavior.^54^ Furthermore, as shown in Figure 5h,i, administration of exenatide solution (twice daily SC), ELPM_SE (single SC), and ELPM_SFEE (single SC) significantly decreased food intake and weight gain in STZ‐induced diabetic mice compared to the control group administered empty PLGA microspheres. The reduction in food intake and weight gain was the greatest in the ELPM_SFEE group, and the order of these two PD effects was as follows: ELPM_SFEE > ELPM_SE > exenatide solution > blank PLGA microspheres.

Based on the results of PD therapeutic efficacy studies, ELPM_SFEE had the most desirable in vivo efficacy for decreasing blood glucose levels, weight gain, and food intake.^52^

## 4 CONCLUSION

In this study, a BBD with four factors and three levels was applied to investigate the effect of process parameters on the fabrication of ELPMs by SFEE. The four process parameters selected as independent variables were pressure (*X*_1_), temperature (*X*_2_), stirring rate (*X*_3_), and flow ratio (*X*_4_), and the effects of independent variables on five responses, including particle size and its distribution (SPAN value), EE, IBR, and residual organic solvent, were elucidated using BBD. Based on experimental results within the design space, a desirable range area for the combination of various variables in the SFEE process was determined through graphical optimization. Exenatide‐loaded PLGA microspheres, obtained from the center point in the design space that satisfied all the response criteria, were compared with PLGA microspheres prepared using a conventional SE method, through various solid‐state characterization assays as well as in vitro and in vivo evaluations. ELPM_SFEE showed improved properties, including a smaller particle size and SPAN value, higher EE, lower IBR, and lower residual solvent. Further, the PK and PD study results indicate better in vivo SR properties and desirable in vivo efficacy, including declined blood glucose levels, weight gain, and food intake, for ELPM_SFEE when compared with those of ELPM_SE. Overall, these results show that SFEE optimization could improve the potential drawbacks of conventional technologies such as the SE process in preparing SR injectable PLGA microspheres. Furthermore, this SFEE technology can be an innovative technology using a SCF that can suggest a solution to the problem of commercial ELPM product in clinical practice with improved PK and PD properties. For the desirable translation of these research results into practical clinical applications, it is essential that the successful scale‐up to a commercial SR formulation production process be completed based on the fundamental understanding of the SFEE process obtained through this DOE study. Therefore, as a further study, we plan to conduct a scale‐up study of this SFEE process.

## AUTHOR CONTRIBUTIONS

**Heejun Park:** Conceptualization (lead); data curation (equal); formal analysis (equal); funding acquisition (equal); investigation (lead); methodology (lead); writing – original draft (lead); writing – review and editing (equal). **Ha Eun‐Sol:** Data curation (lead); formal analysis (lead); methodology (equal); visualization (lead); writing – original draft (supporting); writing – review and editing (equal). **Jeong‐Soo Kim:** Conceptualization (supporting); formal analysis (supporting); investigation (supporting); methodology (supporting); writing – original draft (supporting); writing – review and editing (supporting). **Min‐Soo Kim:** Conceptualization (lead); funding acquisition (lead); investigation (lead); project administration (lead); supervision (lead); writing – original draft (supporting); writing – review and editing (lead).

## CONFLICT OF INTEREST

The authors declare no conflicts of interest.

### PEER REVIEW

The peer review history for this article is available at https://publons.com/publon/10.1002/btm2.10485.

## Supporting information


**Table S1.** All statistical analysis data for model fitting.

## Data Availability

The data that supports the findings of this study are available in the supplementary material of this article.
