# Supplementary material for: Injectable sustained‐release poly(lactic‐co‐glycolic acid) (PLGA) microspheres of exenatide prepared by supercritical fluid extraction of emulsion process based on a design of experiment approach
Source: Bioeng Transl Med. 2023 Jan 2;8(3):e10485. doi: 10.1002/btm2.10485 (PMC10189459; doi:10.1002/btm2.10485)
Supplement: Supplementary file 1 — Table S1. All statistical analysis data for model fitting. [file BTM2-8-e10485-s001.docx]

Injectable sustained-release PLGA microspheres of exenatide prepared by supercritical fluid extraction of emulsion process based on a design of experiment approach

Heejun Park ^1,†^, Eun-Sol Ha ^2,†^, Jeong-Soo Kim ^3^, Min-Soo Kim ^2,*^

^1^ College of Pharmacy, Duksung Women’s University, 33, Samyangro 144-gil, Dobong-gu, Seoul 01369, Korea

^2^ College of Pharmacy, Pusan National University, 63 Busandaehak-ro, Geumjeong-gu, Busan 46241, Korea

^3^ Dong-A ST Co. Ltd., 21, Geumhwa-ro 105beon-gil, Giheung-gu, Yongin-si, Gyeonggi-do 17073, Korea

^†^Heejun Park and Eun-Sol Ha have contributed equally to this work.

E-mail: [minsookim@pusan.ac.kr](mailto:minsookim@pusan.ac.kr)

Table S.1. All statistical analysis data for model fitting

| ANOVA | | | | | | |  | Coefficients in Terms of Coded Factors | | |
| --- | --- | --- | --- | --- | --- | --- | --- | --- | --- | --- |
| Source | Sum of Squares | Df | Mean Square | F-value | *p*-value |  |  | Factor | Coefficient Estimate | Standardized main effect (SME) |
| *Y_1_: Particle size (µm)* | | | | | | |  |  |  |  |
| **Model** | 129.85 | 3 | 43.28 | 12.9 | < 0.0001 | *significant* |  | Intercept | 25.78 | 73.11 |
| X_2_-Temperature | 27.91 | 1 | 27.91 | 8.32 | 0.0084 |  |  | X_2_-Temperature | 1.53 | 2.89 |
| X_3_-Stirring rate | 71.54 | 1 | 71.54 | 21.32 | 0.0001 |  |  | X_3_-Stirring rate | -2.44 | -4.61 |
| X_4_-Flow ratio | 30.4 | 1 | 30.4 | 9.06 | 0.0062 |  |  | X_4_-Flow ratio | -1.59 | -3.01 |
| **Residual** | 77.19 | 23 | 3.36 |  |  |  |  |  |  |  |
| Lack of Fit | 70.93 | 21 | 3.38 | 1.08 | 0.5885 | *not significant* |  |  |  |  |
| Pure Error | 6.26 | 2 | 3.13 |  |  |  |  |  |  |  |
| **Cor Total** | 207.04 | 26 |  |  |  |  |  |  |  |  |
| *Y_2_: SPAN* | | | | | | |  |  |  |  |
| **Model** | 0.5925 | 8 | 0.0741 | 16.92 | < 0.0001 | *significant* |  | Intercept | 1.21 | 31.68 |
| X_1_-Pressure | 0.056 | 1 | 0.056 | 12.8 | 0.0021 |  |  | X_1_-Pressure | 0.0683 | 3.58 |
| X_2_-Temperature | 0.1752 | 1 | 0.1752 | 40.03 | < 0.0001 |  |  | X_2_-Temperature | 0.1208 | 6.32 |
| X_3_-Stirring rate | 0.1776 | 1 | 0.1776 | 40.58 | < 0.0001 |  |  | X_3_-Stirring rate | -0.1217 | -6.37 |
| X_4_-Flow ratio | 0.052 | 1 | 0.052 | 11.88 | 0.0029 |  |  | X_4_-Flow ratio | -0.0658 | -3.45 |
| X_1_² | 0.0284 | 1 | 0.0284 | 6.48 | 0.0203 |  |  | X_1_² | 0.0729 | 2.55 |
| X_2_² | 0.0822 | 1 | 0.0822 | 18.79 | 0.0004 |  |  | X_2_² | 0.1242 | 4.34 |
| X_3_² | 0.0907 | 1 | 0.0907 | 20.73 | 0.0002 |  |  | X_3_² | 0.1304 | 4.56 |
| X_4_² | 0.0142 | 1 | 0.0142 | 3.25 | 0.0881 |  |  | X_4_² | 0.0517 | 1.81 |
| **Residual** | 0.0788 | 18 | 0.0044 |  |  |  |  |  |  |  |
| Lack of Fit | 0.0775 | 16 | 0.0048 | 7.65 | 0.1216 | *not significant* |  |  |  |  |
| Pure Error | 0.0013 | 2 | 0.0006 |  |  |  |  |  |  |  |
| **Cor Total** | 0.6713 | 26 |  |  |  |  |  |  |  |  |
| *Y_3_: Encapsulation efficiency (EE, %)* | | | | | | |  |  |  |  |
| **Model** | 2647 | 9 | 294.11 | 11.52 | < 0.0001 | *significant* |  | Intercept | 1.21 | 29.40 |
| X_1_-Pressure | 5.04 | 1 | 5.04 | 0.1975 | 0.6623 |  |  | X_1_-Pressure | 0.0683 | 0.44 |
| X_2_-Temperature | 138.31 | 1 | 138.31 | 5.42 | 0.0326 |  |  | X_2_-Temperature | 0.1208 | -2.32 |
| X_3_-Stirring rate | 116.31 | 1 | 116.31 | 4.55 | 0.0477 |  |  | X_3_-Stirring rate | -0.1217 | 2.13 |
| X_4_-Flow ratio | 152.65 | 1 | 152.65 | 5.98 | 0.0257 |  |  | X_4_-Flow ratio | -0.0658 | 2.45 |
| X_1_X_2_ | 295.15 | 1 | 295.15 | 11.56 | 0.0034 |  |  | X_1_X_2_ | 0.0729 | -3.40 |
| X_1_² | 1857.54 | 1 | 1857.54 | 72.74 | < 0.0001 |  |  | X_1_² | 0.1242 | -8.52 |
| X_2_² | 269.8 | 1 | 269.8 | 10.57 | 0.0047 |  |  | X_2_² | 0.1304 | -3.25 |
| X_3_² | 520.61 | 1 | 520.61 | 20.39 | 0.0003 |  |  | X_3_² | 0.0517 | -4.51 |
| X_4_² | 234.79 | 1 | 234.79 | 9.19 | 0.0075 |  |  | X_4_² | 1.21 | -3.03 |
| **Residual** | 434.11 | 17 | 25.54 |  |  |  |  |  |  |  |
| Lack of Fit | 424.99 | 15 | 28.33 | 6.21 | 0.1472 | *not significant* |  |  |  |  |
| Pure Error | 9.12 | 2 | 4.56 |  |  |  |  |  |  |  |
| **Cor Total** | 3081.1 | 26 |  |  |  |  |  |  |  |  |
| *Y_4_: Initial burst release (IBR, %)* | | | | | | |  |  |  |  |
| **Model** | 274.05 | 9 | 30.45 | 28.69 | < 0.0001 | *significant* |  | Intercept | 8.48 | 14.26 |
| X_1_-Pressure | 2.81 | 1 | 2.81 | 2.65 | 0.1219 |  |  | X_1_-Pressure | 0.4842 | 1.63 |
| X_2_-Temperature | 0.143 | 1 | 0.143 | 0.1347 | 0.7181 |  |  | X_2_-Temperature | 0.1092 | 0.37 |
| X_3_-Stirring rate | 38.92 | 1 | 38.92 | 36.66 | < 0.0001 |  |  | X_3_-Stirring rate | 1.8 | 6.05 |
| X_4_-Flow ratio | 7.44 | 1 | 7.44 | 7.01 | 0.0169 |  |  | X_4_-Flow ratio | -0.7875 | -2.65 |
| X_1_X_2_ | 18.32 | 1 | 18.32 | 17.26 | 0.0007 |  |  | X_1_X_2_ | 2.14 | 4.15 |
| X_1_² | 200.27 | 1 | 200.27 | 188.69 | < 0.0001 |  |  | X_1_² | 6.13 | 13.74 |
| X_2_² | 33.68 | 1 | 33.68 | 31.73 | < 0.0001 |  |  | X_2_² | 2.51 | 5.63 |
| X_3_² | 23.03 | 1 | 23.03 | 21.7 | 0.0002 |  |  | X_3_² | 2.08 | 4.66 |
| X_4_² | 8.98 | 1 | 8.98 | 8.46 | 0.0098 |  |  | X_4_² | 1.3 | 2.91 |
| **Residual** | 18.04 | 17 | 1.06 |  |  |  |  |  |  |  |
| Lack of Fit | 16.96 | 15 | 1.13 | 2.09 | 0.3713 | *not significant* |  |  |  |  |
| Pure Error | 1.08 | 2 | 0.5414 |  |  |  |  |  |  |  |
| **Cor Total** | 292.09 | 26 |  |  |  |  |  |  |  |  |
| *Y_5_: Residual solvent (ppm)* | | | | | | |  |  |  |  |
| **Model** | 83758.28 | 5 | 16751.66 | 34.22 | < 0.0001 | *significant* |  | Intercept | 100.17 | 13.59 |
| X_1_-Pressure | 28714.08 | 1 | 28714.08 | 58.66 | < 0.0001 |  |  | X_1_-Pressure | -48.92 | -7.66 |
| X_2_-Temperature | 17980.02 | 1 | 17980.02 | 36.73 | < 0.0001 |  |  | X_2_-Temperature | -38.71 | -6.06 |
| X_4_-Flow ratio | 32354.47 | 1 | 32354.47 | 66.1 | < 0.0001 |  |  | X_4_-Flow ratio | -51.93 | -8.13 |
| X_1_² | 1846.2 | 1 | 1846.2 | 3.77 | 0.0657 |  |  | X_1_² | 16.98 | 1.94 |
| X_4_² | 3723.94 | 1 | 3723.94 | 7.61 | 0.0118 |  |  | X_4_² | 24.12 | 2.76 |
| **Residual** | 10278.95 | 21 | 489.47 |  |  |  |  |  |  |  |
| Lack of Fit | 10205.01 | 19 | 537.11 | 14.53 | 0.0663 | *not significant* |  |  |  |  |
| Pure Error | 73.94 | 2 | 36.97 |  |  |  |  |  |  |  |
| **Cor Total** | 94037.23 | 26 |  |  |  |  |  |  |  |  |
